# Advanced Imaging Strategies Based on Intelligent Micro/Nanomotors

**DOI:** 10.34133/cbsystems.0384

**Published:** 2025-09-10

**Authors:** Dang Zhang, Liang Lin, Chao Deng, Mohamed Syazwan Osman, Paul E.D. Soto Rodriguez, Fei Han, Mingyu Li, Lei Wang

**Affiliations:** ^1^ State Key Laboratory of Advanced Inorganic Fibers and Composites, School of Chemistry and Chemical Engineering, Harbin Institute of Technology, Harbin 150001, China.; ^2^ Wenzhou Jiayuan Biotechnology Co., Ltd., Wenzhou 325000, China.; ^3^ College of Chemistry and Materials Engineering, Wenzhou University, Wenzhou 325027, China.; ^4^ EMZI-UiTM Nanoparticles Colloids & Interface Industrial Research Laboratory (EMZI NANO-CORE), Faculty of Chemical Engineering, Universiti Teknologi MARA, Cawangan Pulau Pinang, 13500 Permatang Pauh, Pulau Pinang, Malaysia.; ^5^ Instituto de Estudios Avanzados IUDEA, Departamento de Física, Universidad de La Laguna C/Astrofísico Francisco Sánchez, s/n.E-38203 Tenerife, Spain.; ^6^ School of Science, Wuhan University of Technology, Wuhan 430070, China.

## Abstract

Biological imaging has revolutionized tissue analysis by revealing morphological and physiological dynamics, yet faces inherent limitations in penetration depth and resolution. Micro/nanomotors (MNMs), with autonomous propulsion and spatiotemporal control, offer transformative solutions to traditional static imaging paradigms. These dynamic contrast agents enhance detection sensitivity in ultrasound, fluorescence, photoacoustic, and magnetic resonance imaging via motion-amplified signal modulation, enabling real-time tracking of subcellular events and microenvironmental changes. While MNMs-enhanced bioimaging has advanced rapidly, systematic analysis of their mechanisms and challenges remains limited. Based on our research experience in this field, this paper first summarizes the signal-enhancing mechanisms of MNMs in single-modal imaging. It then explores multimodal applications through MNMs-probe design and discusses artificial intelligence-driven intelligent MNMs for precision imaging. Finally, challenges and outlook are outlined, aiming to provide a theoretical framework and research roadmap for MNMs-mediated bioimaging technologies.

## Introduction

Biological imaging technology has emerged as a direct technique for visualizing and acquiring multidimensional information about organisms. This technology offers essential tools for examining the structure, morphology, and functionality of targeted regions at the organ, tissue, and cellular scales. Currently, a variety of imaging techniques, including magnetic resonance imaging (MRI) [1], ultrasound imaging (USI) [2], fluorescence imaging (FLI) [3], have been established. These methods utilize external stimuli, including electromagnetic radiation, sound, magnetism, optoelectronics, and molecular probes, to interact with the target samples. Informational feedback from these interactions provides biological data at varying scales and spatial resolutions. While demonstrating diagnostic utility, current imaging platforms retain fundamental constraints requiring advancement in spatiotemporal resolution, real-time tracking fidelity, and multimodality integration within intricate biological matrices. FLI, for example, is limited by weak tissue penetration and susceptibility to photobleaching. MRI depends on high concentrations of contrast agents and does not offer adequate spatial resolution.

Contemporary bioimaging systems face dual challenges of constrained spatial resolution and tissue penetration depth, frequently requiring exogenous contrast agents for diagnostic visualization [4]. Nevertheless, conventional nanoparticle-based agents demonstrate suboptimal biodistribution profiles, exhibiting nontargeted diffusion, rapid clearance, and off-target deposition. Multiscale photon propagation dynamics, particularly through coherent scattering processes and inelastic absorption mechanisms, mediate stochastic signal degradation that fundamentally limits achievable spatial resolution in deep-tissue imaging modalities [5]. These limitations collectively hinder precision diagnostics and therapeutic monitoring, driving demand for engineered contrast systems with enhanced targeting specificity and signal-to-noise ratio in complex biological matrices.

Recent advancements in micro/nanomotors (MNMs) technology present promising solutions to the challenges encountered in bioimaging techniques. These motors, which convert exogenous energy such as magnetic [6,7], electric [8], light [9,10], sound [11], or chemical energy [12–14] into mechanical motion, have emerged as a new class of intelligent nanomaterials. Owing to their unique autonomous movement, precise targeting capabilities, and integrated multifunctional features, MNMs offer innovative prospects for environmental remediation [15,16], medical disease diagnosis [17–21], drug delivery [22–25], and especially for cancer therapy [26–30]. When used as innovative imaging agents, these motors can overcome the limitations of traditional passive diffusion agents, enabling dynamic contrast agent delivery, real-time signal tracking, and synergy in multimodal imaging within complex biological environments. MNMs have the potential to serve as active contrast agents, penetrating biological barriers such as the blood–brain barrier, mucus layer, and cell membrane [31–33]. This ability enables them to concentrate contrast agents in lesion areas, thereby substantially increasing the depth of deep-tissue imaging. Additionally, these motors can control the aggregation position in blood vessels or tissues through targeted navigation [34–36]. They release imaging agents under specific microenvironmental conditions at the target location, which helps reduce imaging background noise and enhance imaging resolution. Furthermore, the unique physicochemical properties of micro and nano materials, including surface plasmon resonance (SPR) [37,38], aggregation-induced emission (AIE) enhancement fluorescence [39], and nanozyme catalytic reactions [40,41], can be leveraged by these motors to amplify imaging signals. This results in high-contrast imaging of target sites. Recently, notable advancements have been achieved in localizing and identifying MNMs through the integration of deep learning (DL) and imaging techniques. DL algorithms enable automatic detection and extraction of MNMs imaging features even in complex backgrounds [42,43].

**Fig. 1.**
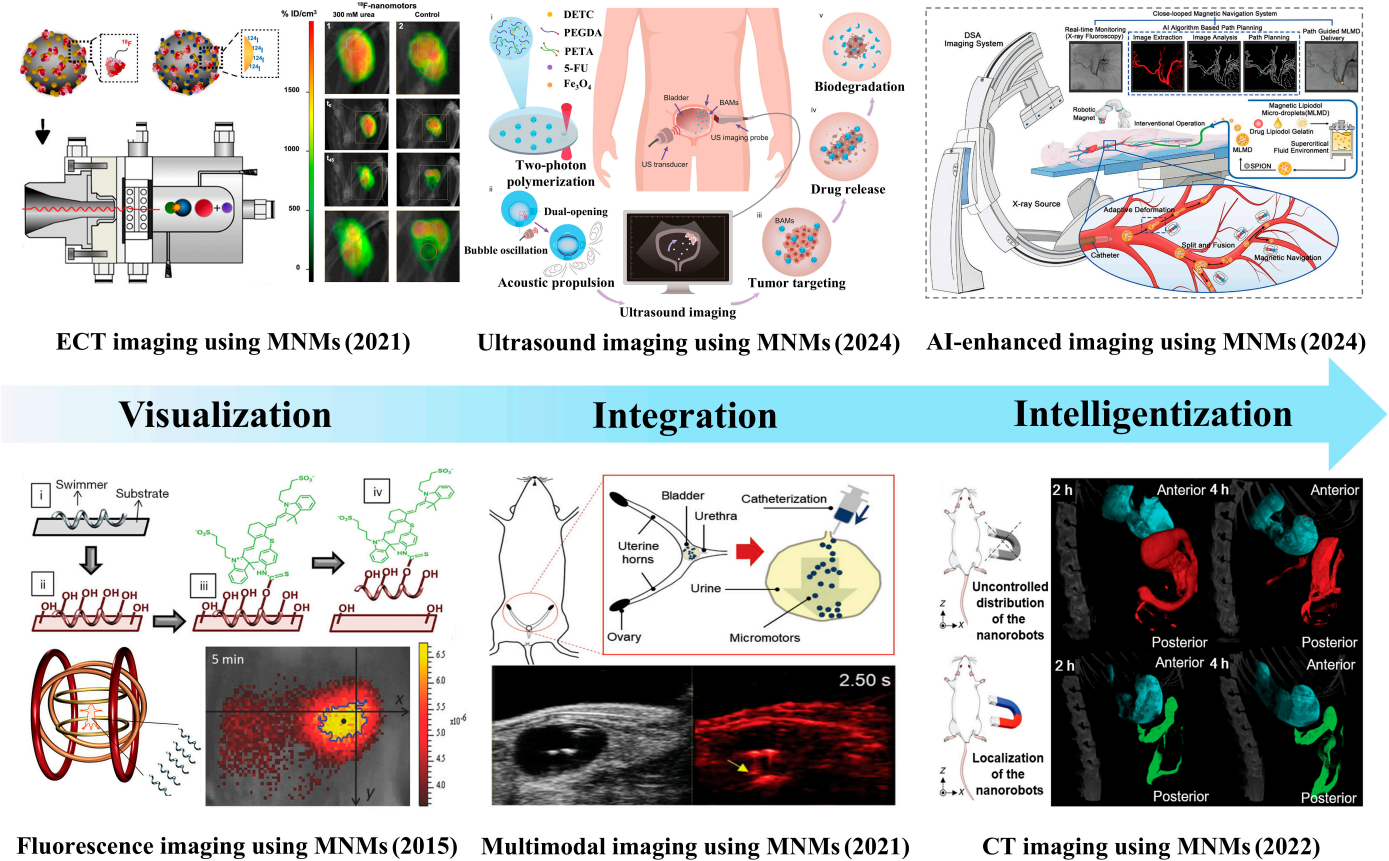
A brief summary of development history of imaging based on micro/nanomotors (MNMs) and some typical examples. Schematic illustration source: Fluorescence imaging using MNMs [57], Copyright 2015 Wiley-VCH GmbH. ECT imaging using MNMs [118], Copyright 2021 AAAS. Ultrasound imaging using MNMs [86], Copyright 2024 AAAS. Multimodal imaging using MNMs [132], Copyright 2021 Wiley-VCH GmbH. AI-enhanced imaging using MNMs [140], Copyright 2024 Wiley-VCH GmbH. CT imaging using MNMs [111], Copyright 2022 Wiley-VCH GmbH.

MNMs, serving as innovative contrast agents, present a disruptive solution to the traditional trade-off between spatial resolution and tissue penetration depth in bioimaging, as shown in Fig. 1. Current research predominantly centers on tracing the in vivo motion trajectory of these motors [44–47], neglecting their potential role as active contrast-enhancing media, especially their associated enhancement mechanisms. Accordingly, drawing on prior experience in the field, this study presents a systematic analysis of the functional mechanisms by which MNMs operate as contrast agents within conventional imaging modalities. Subsequent sections explore recent advancements in their applications for multimodal imaging and integration with artificial intelligence (AI)-driven technologies. Finally, the potential opportunities and challenges underpinning the future development of MNMs-enhanced bioimaging are discussed, with the aim of establishing a foundational understanding to facilitate translational progress from laboratory research to clinical implementation.

## The Advantages of MNMs as Biological Imaging Agents

**Fig. 2.**
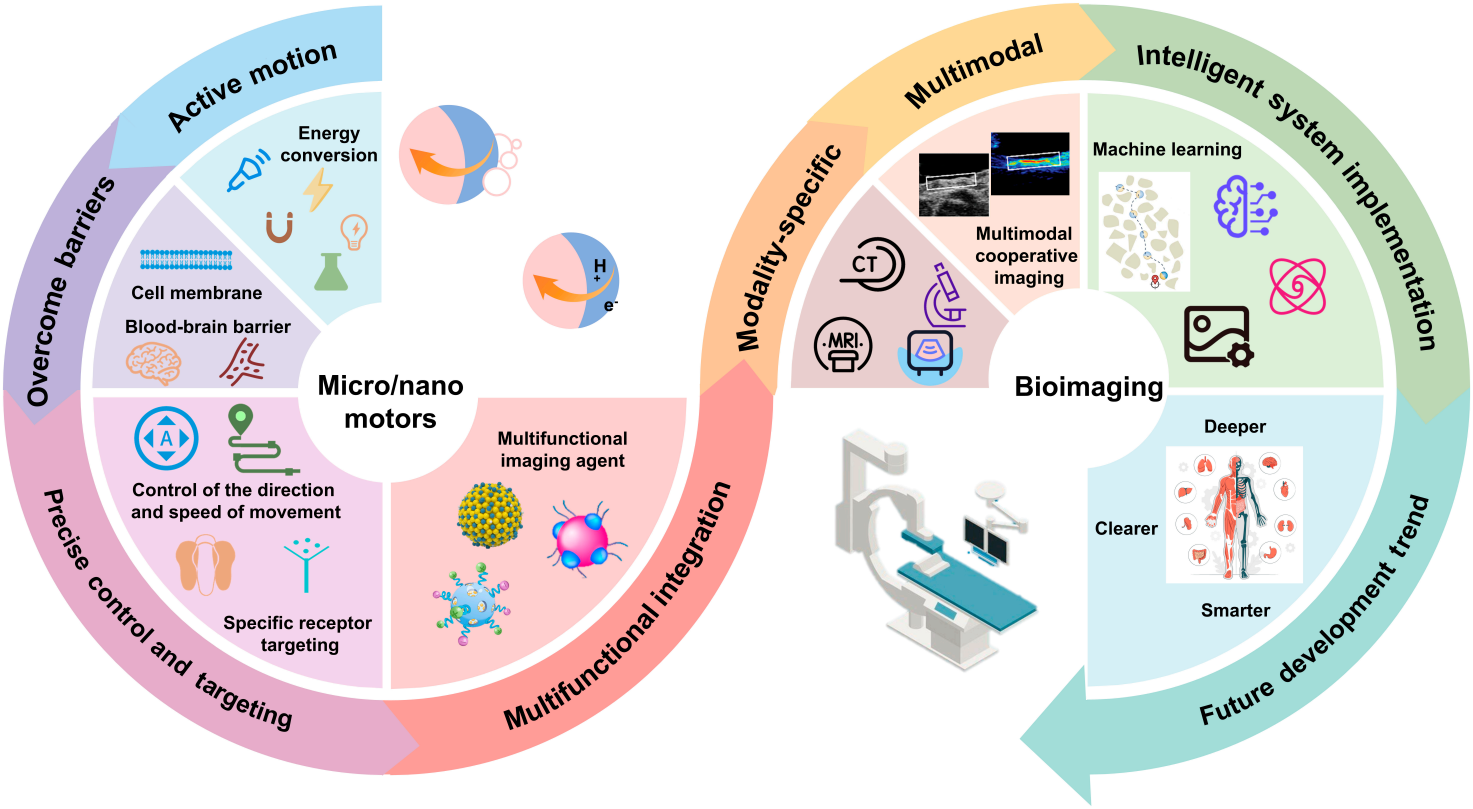
Schematic illustration of MNMs for bioimaging.

MNMs exhibit obvious advantages and unique characteristics compared to traditional micro- or nanoparticles, making them especially promising for applications in biological imaging. The specific advantages and enhanced imaging strategies are summarized in Fig. 2.

### Active motion

Traditional imaging agents rely on passive diffusion or blood circulation for in vivo distribution, resulting in suboptimal imaging kinetics and vulnerability to biological barriers. By contrast, MNMs convert external energy into autonomous motion, enabling active navigation toward target regions. This motility markedly enhances imaging efficiency by facilitating rapid accumulation in the target area. Additionally, their active movement promotes prolonged interaction with biological tissues/cells, increasing probe concentration and retention time to amplify imaging signals, thereby improving sensitivity and resolution [48]. Moreover, MNMs generate local circulations by driving surrounding fluids/cells, altering the physicochemical environment to boost imaging contrast and clarity.

### Overcoming biological barriers

Owing to their nanoscale dimensions and autonomous motility, MNMs can traverse complex biological barriers—such as the blood–brain barrier and cell membranes—and access deep tissues and cells inaccessible to traditional imaging agents. Their active motility and deformability enable trans-membrane entry, facilitating intracellular imaging and providing a robust platform for studying cellular physiology and pathology. High-speed motility allows them to overcome bloodstream shear forces in vascular systems. Such environmental adaptability ensures effective imaging across diverse anatomical niches, addressing limitations in traditional agent distribution and tissue penetration.

### Precise control and targeting

Precise control over driving parameters, including intensity, direction, and frequency, enables accurate positioning and navigation of MNMs, allowing them to follow preprogrammed trajectories and reach targeted imaging sites [49]. For instance, magnetic MNMs can be steered by external magnetic fields to migrate toward specific tissues or organs, enabling localized precision imaging. During imaging, their motion and positioning can be real-time adjusted to track dynamic biological processes, facilitating detailed observation of in vivo changes. This capability provides high-fidelity data for early disease diagnosis and treatment efficacy evaluation, addressing limitations in static imaging techniques.

### Multifunctional integration

MNMs act as multifunctional platforms for integrating diverse imaging modalities, including fluorescence, magnetic resonance, and ultrasound, to enable multimodal diagnostics. Surface conjugation of fluorescent dyes, magnetic nanoparticles (MNPs), or other contrast agents allows these motors to facilitate simultaneous multimodal imaging, providing clinicians with comprehensive diagnostic insights. Beyond imaging, they can encapsulate therapeutic payloads such as drugs or genetic materials, enabling theranostic integration: upon reaching target sites, stimuli-responsive MNMs release cargo in response to biological cues (e.g., pH and temperature), achieving precise therapy while enabling real-time imaging monitoring of treatment efficacy. This dual functionality bridges diagnostic imaging and targeted therapy, addressing critical gaps in precision medicine.

## Modality-Specific Enhancement Approaches Based on MNMs

MNMs substantially improve medical imaging resolution, contrast, and dynamic tracking via self-propulsion, targeted accumulation, and cargo loading. This section will elaborate on the enhancement mechanisms of various imaging modalities based on MNMs individually.

### Fluorescence imaging

**Fig. 3.**
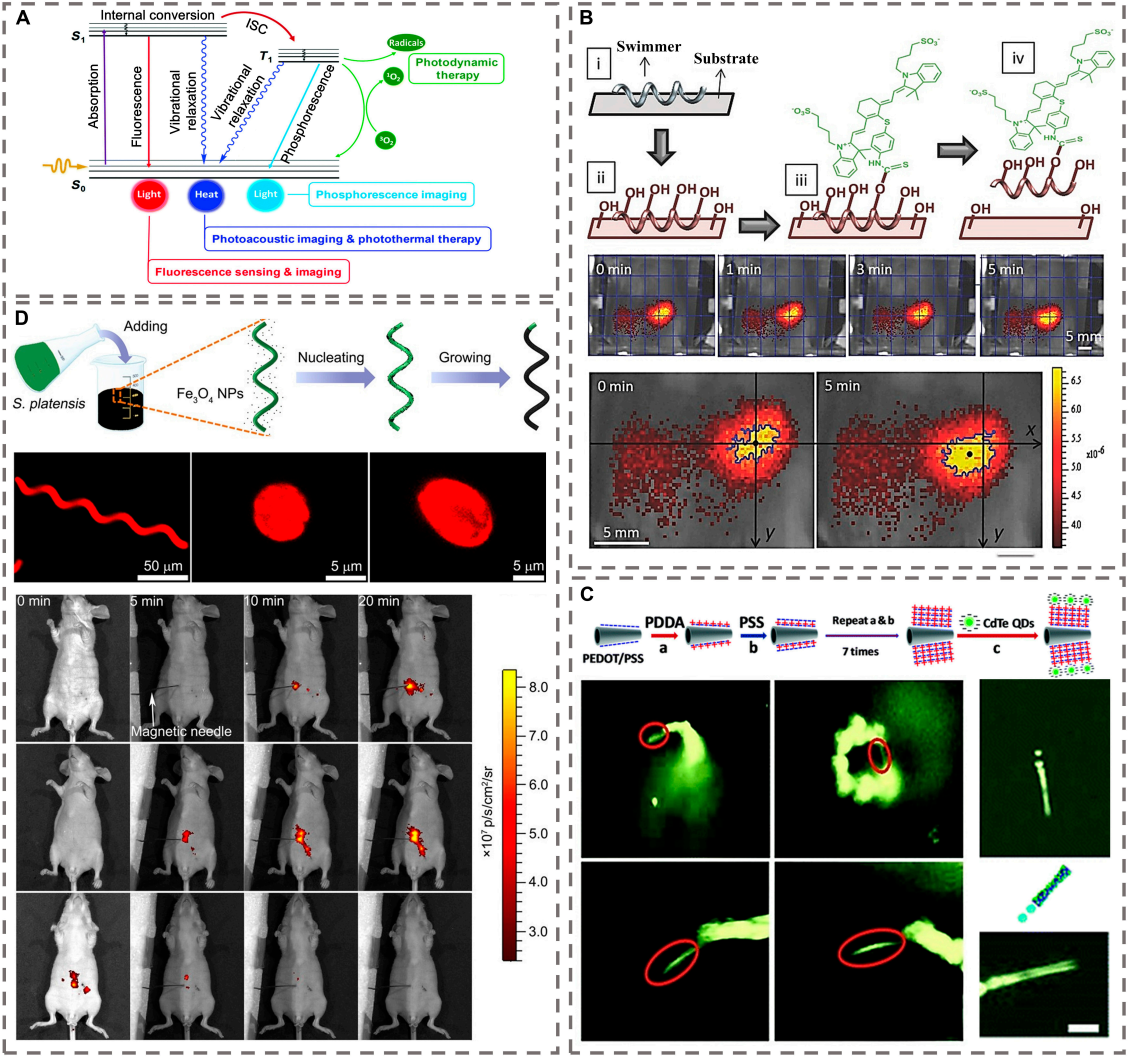
Schematic illustration of the formation and fluorescence imaging of MNMs. (A) Schematic of a Jablonski diagram. Reproduced from Ref. [52], Copyright 2020 RSC. (B) Fabrication and fluorescent pictures of MNMs with NIR-797 dyes. Reproduced from Ref. [57], Copyright 2015 Wiley-VCH GmbH. (C) Preparation and fluorescent pictures of QD-based MNMs. Reproduced from Ref. [63], Copyright 2015 RSC. (D) Preparation and fluorescent pictures of bioautofluorescent MNMs. Reproduced from Ref. [70], Copyright 2017 AAAS.

FLI is widely employed for visualizing biological tissues and cells, playing a critical role in biomarker tracking, medical diagnostics, and related fields [50,51]. The performance of FLI technology depends on the inherent characteristics of fluorescent substances. When excited by a specific energy quantum, electrons in fluorophore materials transition from the ground state (*S*_0_) to a higher-energy singlet state (*S*_1_). Due to vibrational/rotational energy differences, electrons first relax within the *S*_1_ state via nonradiative decay before emitting lower-energy photons upon returning to *S*_0_. This process necessitates excitation energy that is at least equal to the singlet–ground state energy gap, (Δ*E* = *ES*_1_ *− ES*_0_), as illustrated in Fig. 3A [52]. While organic dyes, quantum dots (QDs), and other fluorescent agents have enabled substantial advancements, their passive diffusion in complex physiological environments often leads to suboptimal target accumulation and imaging contrast. MNMs address this limitation by enabling active transport of fluorescent payloads: fluorescent materials can be covalently conjugated, physically adsorbed, or integrated as the structural matrix of MNMs.

#### Fluorescent functionalization-enabled imaging enhancement based on MNMs

Organic fluorescent dyes are among the most widely used fluorophores due to their small size and low steric hindrance, enabling covalent conjugation to MNMs via chemical modification [53]. In 2015, Singh et al. [54] prepared fluorescent nanomotors by grafting fluorescein amine onto silica particles. The nanomotors were driven by oxygen bubbles generated by Pt-catalyzed hydrogen peroxide. It was demonstrated that stable fluorescent signals could be detected in aqueous media, proving the possibility of fluorescent nanomotors for FLI. A similar attempt was made by Mhanna et al. [55]. They prepared artificial bacterial flagellar MNMs functionalized with water-soluble calcium xanthophyll fluorophores. The results demonstrated that the fluorophores were able to emit fluorescent signals stably even after binding to the nanomotors, suggesting their potential for FLI. However, their short-wavelength signals limit in vivo use due to poor tissue penetration.

Leveraging the deep-tissue penetration of near-infrared (NIR) light [56], the same group later replaced these dyes with the NIR probe NIR-797. As shown in Fig. 3B [57], the nanomotors were modified by a nickel layer based on the structure of bacterial flagella, which allowed the motor to move at 70.4 μm/s when exposed to the magnetic field. In addition, to realize in vivo motion for FLI, its biocompatibility was enhanced by coating the Ti layer. The results of the trial revealed that the fluorescence-modified nanomotor has an excellent capability for targeted imaging. For intracellular imaging, Lin et al. [58] prepared NIR light-driven nanomotors using fluorophore carboxyfluorescein (FAM) labeling. The nanomotor was based on mesoporous silica as the core, coated with a gold layer and an MnO_2_ nanolayer on both sides. The gold layer was used to generate a thermal gradient by infrared light irradiation, while the MnO_2_ nanolayer was used to load hairpin DNA quadrangular nanostructure (hQN) probes. Motor motion enhanced hQN probe binding to target miRNAs, triggering catalytic hairpin assembly reactions for amplified fluorescence. Yang et al. [59] extended this concept using Janus DNA motors dual-labeled with FAM/Cy5, enabling simultaneous imaging of 2 miRNAs via asymmetric structure-induced signal separation. They labeled FAM and Cy5 fluorophores on both sides of the Janus structure respectively and successfully realized FLI of 2 different miRNAs. The asymmetric structure of the nanomotors efficiently reduced the fluorescence signal interference and increased the immobilized concentration of the 2 different signaling probes, which substantially enhanced the fluorescence intensity.

Despite these advances, organic dyes suffer from rapid photobleaching and autofluorescence interference. Indocyanine green (ICG) addresses these issues through its NIR-II fluorescence (1,000 to 1,700 nm), integrated into polydopamine-shelled PS@PDA-ICG nanomotors [60]. Upon exposure to NIR-I light, the nanomotors align, inducing photothermal effects that propel thermal swimming motion. The NIR-II fluorescence of ICG enables precise tracking of the nanomotor’s directional movement within subcutaneous tissues, such as its aggregation along the light’s direction. This fluorescence also aids in reducing tissue penetration resistance through photothermal softening, thereby enhancing targeting efficiency. Tumor accumulation via thermotaxis increases local dye concentration, reducing quenching and enhancing fluorescence intensity. This design substantially improves subcutaneous imaging resolution, penetration depth, and targeting accuracy.

#### Spectral engineering-enabled imaging enhancement based on MNMs

QDs, nanoscale semiconductor crystals (II-VI/III-V elements), offer superior performance to organic dyes in FLI due to quantum size and dielectric confinement effects [61]. QDs exhibit unique luminescent properties due to the quantum size effect and dielectric confinement effect. Compared to organic dyes with narrow excitation spectra and broad emission spectra, the emission spectra of QDs can be adjusted according to the size and composition of the nuclei of the QDs, allowing them to be excited and emitted in the NIR region, which produces brighter fluorescence with deeper penetration [62]. Furthermore, the wide excitation range of QDs makes it possible to stimulate various hues of QDs with a single excitation wavelength, which raises the possibility of simultaneously identifying multiple fluorophores. With their anti-photobleaching properties, high extinction coefficient, and high quantum yield, QDs demonstrate exceptional potential for FLI.

These properties, combined with strong conjugation ability (e.g., electrostatic adsorption), facilitate integration with MNMs. Jurado-Sánchez et al. [63] first grafted CdTe QDs onto poly(3,4-ethylenedioxythiophene) (PEDOT)/Pt micromotors, demonstrating fluorescence-based motion visualization and laying the groundwork for mobile nanoplatforms. The experimental results showed that the motion behavior of the nanomotor can be observed by fluorescence and demonstrated the potential of fluorescent QDs in combination with mobile-capable nanoplatforms, laying the foundation for FLI, as shown in Fig. 3C. Wu et al. [64] further developed a multifunctional system by encapsulating MNPs, CdTe QDs, and doxorubicin (DOX) in erythrocytes, enabling ultrasonic/magnetic-driven navigation with dual QD/DOX fluorescence for concurrent therapy monitoring. In this system, iron oxide MNP, water-soluble CdTe QD nanocrystals, and chemotherapeutic DOX were loaded into erythrocytes by a hypotonic dilution encapsulation approach. The asymmetric distribution of MNP allowed the MNMs to be driven by ultrasonic fields and enabled efficient magnetic field navigation. Both QD and DOX were able to emit fluorescence for visualization of MNMs. This MNMs multifunctional platform enables different modalities for treating and monitoring diseases simultaneously, which is expected to be the future trend.

**Fig. 4.**
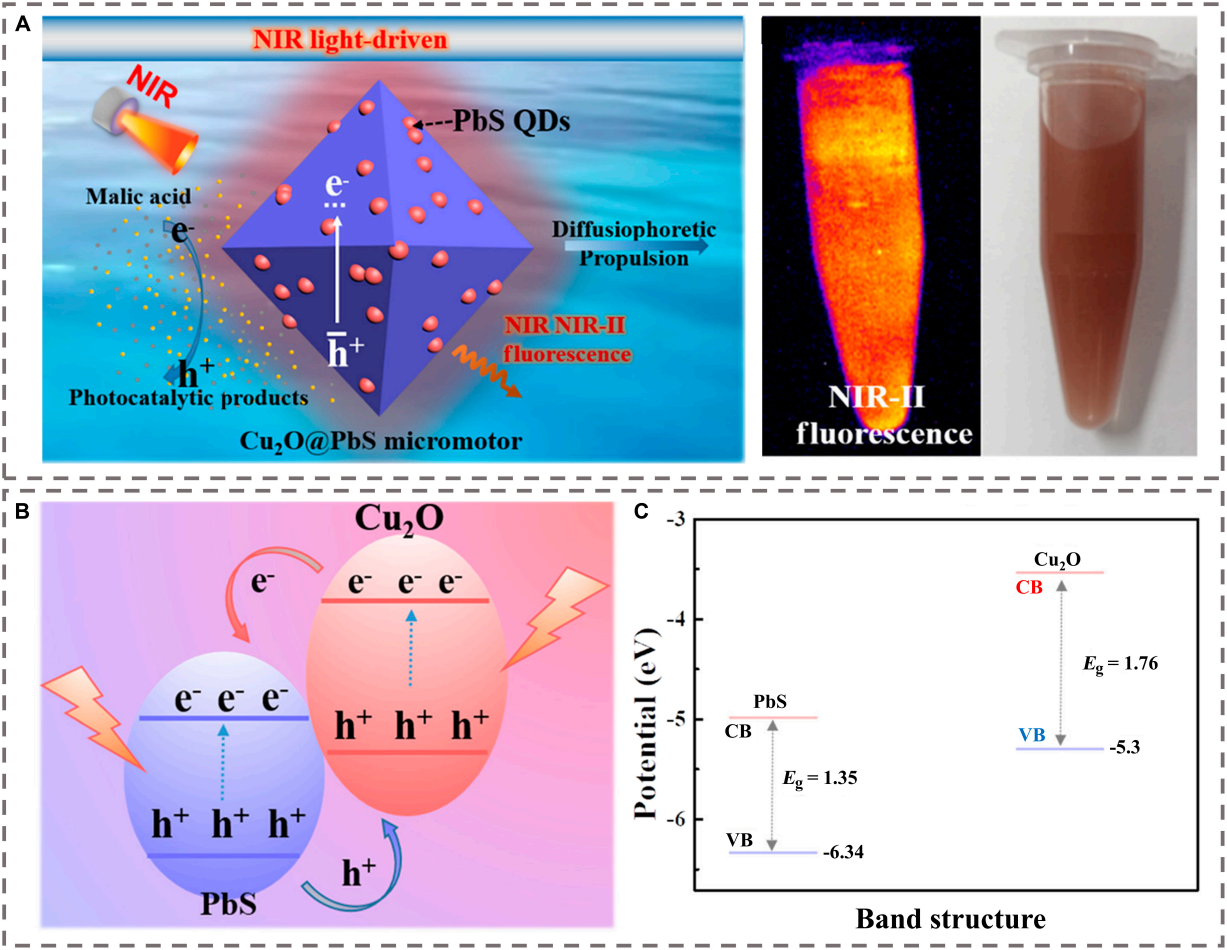
Quantum dot-based micromotors with NIR-I light photocatalytic propulsion and NIR-II fluorescence. Reproduced from Ref. [68], Copyright 2022 American Chemical Society. (A) Propulsion mechanism and NIR-II fluorescence image of Cu_2_O@PbS micromotors with NIR-I light irradiation. (B) The charge transport process of the Cu_2_O@PbS heterojunction under the irradiation of NIR light. (C) Energy band alignments of the Cu_2_O@PbS heterojunction.

However, cytotoxicity of traditional QDs (e.g., Cd-based) restricts in vivo use [65]. Carbon QDs with good biocompatibility have been developed for applications in in vivo imaging [66]. Biocompatible alternatives like graphene QDs (GQDs) have been integrated into Janus motors for dark-field tracking via intense blue fluorescence [67]. The fluorescence properties of QDs within the second NIR light region, when coupled with the efficient propulsion capabilities of MNMs, have further augmented the effectiveness of FLI. Cu₂O@PbS micromotors were synthesized via a one-step hydrothermal process, which involved doping PbS QDs into Cu₂O micromotors, as shown in Fig. 4 [68]. The incorporation of these QDs endowed the micromotors with NIR region II (NIR-II, 1,100 nm) fluorescence properties. This feature allows for label-free real-time tracking, eliminating the need for additional modifications and addressing issues related to imaging contrast and deep-tissue penetration in complex biological environments. Concurrently, the PbS QDs form type II heterojunctions with Cu₂O, expanding the light absorption in the NIR region I (NIL-I, 808 nm) and facilitating charge separation, thereby substantially enhancing photocatalytic efficiency. This enables the micromotors to achieve efficient propulsion, with a maximum speed of 11.86 μm/s, in ultralow-concentration biofuels such as 0.125 mM malic acid.

Although QDs have proven to be promising probes for FLI, the imaging applications of QDs still face some challenges, including intermittent QD fluorescence and unresolved biocompatibility. Future efforts focus on low-toxicity QDs (e.g., carbon-based, III-V materials) to enable safe in vivo applications, combining their optical superiority with motor-driven targeting for advanced FLI.

#### Bioluminescence imaging enhancement based on MNMs

Bioautofluorescent materials hold great promise for imaging guidance due to their relatively excellent biocompatibility [69]. Yan et al. [70] leveraged autofluorescent *Spirulina* as a template, coating Fe₃O₄ nanoparticles via dip-coating to fabricate helical magnetic motors (Fig. 3D). These motors exhibited rapid magnetic-driven motion and homogeneous green fluorescence under excitation, enabling in vivo tumor imaging and apoptosis-inducing cancer therapy in mice. Advancing the development of autofluorescent MNMs-based cancer therapy methods, Zhong et al. [71] prepared magnetic *Spirulina platensis* hybrid MNMs. Superparamagnetic Fe_3_O_4_ nanoparticles were functionalized on *S. platensis*, which was utilized as an actuator. The presence of autofluorescent chlorophyll in *S. platensis* provided substantial support to its in vivo imaging. At 2.5 h after ejection, the tumor site’s fluorescence intensity peaked after increasing progressively. Volvox, rich in chlorophyll, further enhances imaging with MNMs [72]. Simultaneously, the coordinated movement of thousands of flagella on the Volvox surface creates a fluid-mixing effect. This can enhance the local microenvironment, such as the distribution of oxygen and imaging agents, indirectly boosting signal consistency. Despite the fact that most autofluorescent materials can emit the usual blue or green color emission, most bioautofluorescent materials emit short-wavelength (blue/green) light, limiting penetration depth to superficial tissues. Additionally, overlapping tissue autofluorescence creates background interference, necessitating development of NIR-emitting biological templates to expand in vivo applications.

#### AIE-enabled luminescent imaging enhancement based on MNMs

**Fig. 5.**
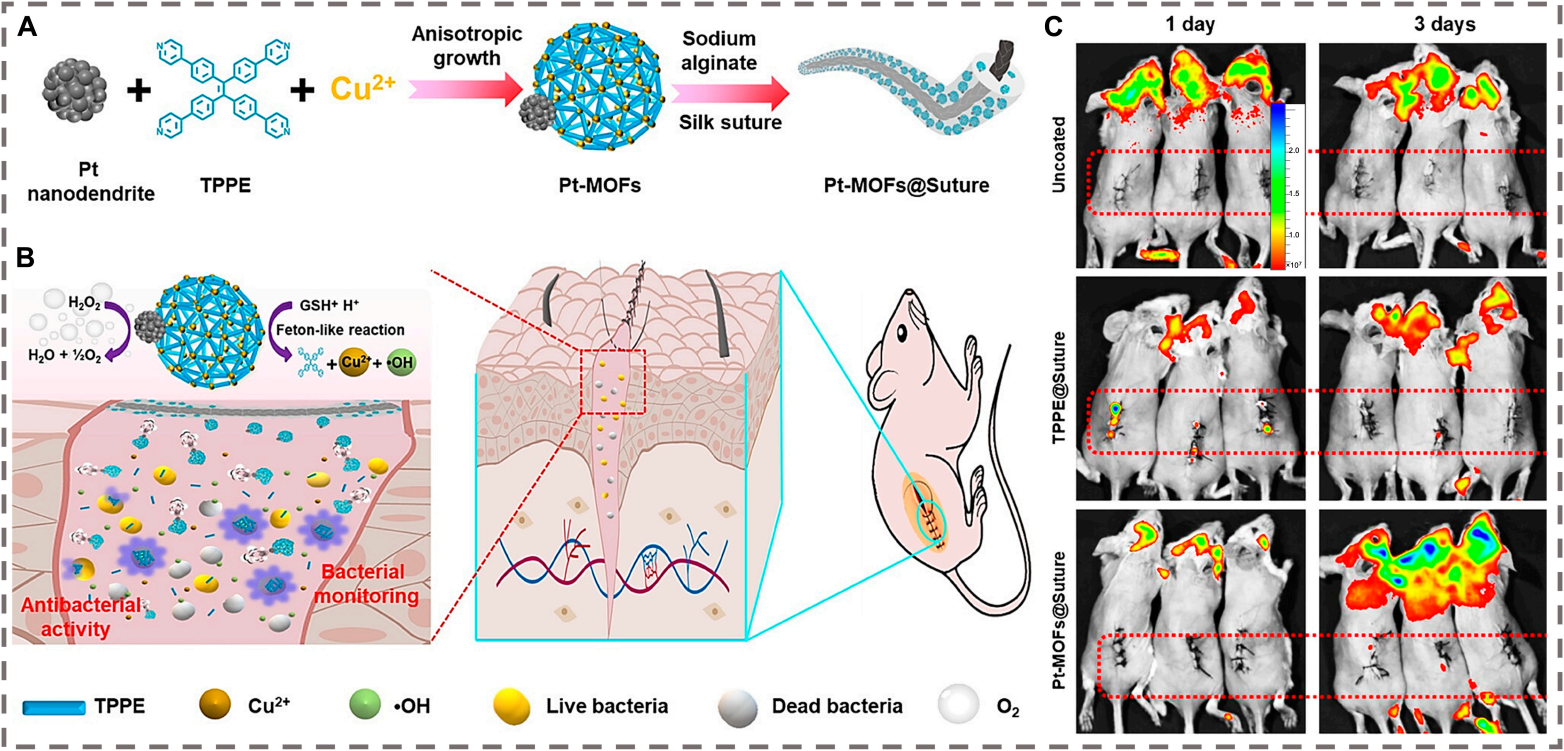
Schematic illustration of the formation and bioimaging of Pt-MOFs nanomotors. (A) Fabrication process of Pt-MOFs nanomotors-coated sutures. (B) Infected microenvironment-activated Pt-MOFs nanomotors for bacterial monitoring and antibacterial activity. (C) Fluorescence images of *S. aureus*-infected surgical wound mice sutured with uncoated suture, TPPE@Suture, and Pt-MOFs@Suture at predetermined time points. Reproduced from Ref. [76], Copyright 2025 Wiley-VCH GmbH.

Recent advancements in deep-tissue FLI leverage AIE molecules, which exhibit intense infrared fluorescence in aggregated states, enabling real-time monitoring of deep cerebrovascular systems [73,74]. The researchers proposed to use AIE moiety-containing polymersomes to provide stable fluorescence for the nanomotors [75]. AIEgenic polymersomes, comprising polyethylene glycol (PEG) and poly(trimethylene carbonate) blocks, provide stable fluorescence for nanomotors modified with peroxidase/urease via layer-by-layer assembly. These motors navigate hydrogen peroxide/urea gradients, with AIE fluorescence activated by restricted intramolecular motion, offering high-quality signals for infection imaging. Pt-MOFs MNMs further enhance bacterial infection imaging through multimechanism synergy, as shown in Fig. 5 [76]. Copper-MOFs embedded with tetrafluoroethylene (TPPE) and platinum nanodendrites respond to infection microenvironments. Pt catalyzes H₂O₂ to generate propulsive oxygen bubbles, improving biofilm penetration, while acidic conditions trigger TPPE protonation and electrostatic adsorption onto bacterial surfaces. Concurrently, glutathione (GSH) reduces Cu^2+^-induced quenching, activating strong blue fluorescence. In vitro/in vivo studies confirm specific gram-positive/negative bacterial imaging and wound infection monitoring, with suture-coated motors accumulating signals to track infection dynamics. This AIE-driven, self-propelled, and microenvironment-responsive design overcomes traditional limitations in penetration and specificity, providing an efficient strategy for real-time infectious disease imaging.

MNMs have substantially advanced FLI , yet challenges persist due to inherent limitations of fluorescent materials, including limited spatial resolution, shallow tissue penetration, and suboptimal biocompatibility. Key hurdles include developing more stable, bright, and biocompatible fluorophores, as well as optimizing imaging performance when motors carry multiple agents or operate in clusters. Additionally, the disparity in imaging efficiency between single and collective motor behavior remains uncharacterized. Future research should prioritize integrating FLI with complementary modalities to overcome these constraints, fostering more comprehensive and clinically applicable diagnostic solutions.

### Ultrasound imaging

USI is a noninvasive bioimaging technique that is promising for the diagnosis of angiogenesis, inflammation, and thrombosis with the advantages of safety, deep penetration, and low cost [77,78]. It generates 2D tomographic images by transmitting acoustic waves via a transducer and processing reflected signals, offering superior temporal resolution for dynamic feedback [79]. MNMs enhance US imaging through dual roles. US-guided navigation facilitates their targeted drug delivery and therapeutic tasks, while integration with modalities like microbubble imaging, B-mode, and Doppler boosts imaging contrast and depth [80]. Additionally, MNM swarming increases effective density, improving deep-tissue bioimaging potential.

#### Microbubble-mediated contrast enhancement by MNMs

The B-mode, or brightness mode, is one of the frequently utilized imaging modes in USI. Grayscale images are obtained by transmitting brief ultrasonic pulses into objects and recording backscattered echoes. Microbubbles are commonly used as contrast agents or echo enhancers. In response to a transmitted acoustic wave, a shelled microbubble resonates, and its linear resonant frequency *f*_r_ is defined by its radius *r*, the polytropic index of gas *κ*, the equilibrium pressure inside the microbubble *p*_e_, the shear modulus of the shell *G*_S_, the density of the surrounding medium *ρ*, and instantaneous shell thickness *d*_s_ [81], as shown in Eq. (1).

$$f_{r}=\frac{1}{2\pi r}\sqrt{\frac{3\kappa p_{e}+12G_{s}\frac{d_{s}}{r}}{\rho }}$$ (1)

Microbubbles emerge nonlinear as the ultrasonic amplitude increases. It reacts favorably to the ultrasonic wave’s fractal pressure but poorly to the wave’s compression pressure. As a result, while scanning, the harmonic frequency components produced by the microbubbles are noticeably greater than those produced by the tissue. Ultrasonic enhanced pictures can be obtained because the microbubbles have a strong echo property at low mechanical indices.

To enhance US imaging sensitivity and targeting, catalytic micromotors decomposing hydrogen peroxide to generate oxygen microbubbles have been developed [82]. The track of micromotors can be detected through ultrasonic imaging based on O_2_ microbubbles created by the decomposition of H_2_O_2_. Due to the presence of a large accumulation of neutrophils around the abscess and elevated levels of H_2_O_2_, the study was successful in USI of micromotors in an in vivo rat abscess model. Similarly, in order to utilize microbubble contrast agents for imaging, Feng et al. [83] reported the use of a Janus Mg/Ni-based micromotor as an ultrasonic contrast agent for medical imaging. Magnesium cores react with ionic environments to produce self-propelling microbubbles, whose gas–liquid acoustic impedance mismatch enhances US contrast. Tunable sizes allow intravenous injection for intravascular angiography via chloride-triggered bubble formation.

Chen et al. [84] designed an ultrasound tracking technique for acoustically driven MNMs in order to build an imaging strategy for in situ applications. This was accomplished through the manufacture of hollow microtubular nanomotors, where the hydrophobicity of the material allowed for the encapsulation of air bubbles in the hollow cavities. Xu et al. successfully accomplished the ultrasonic localization of MNMs within the joint cavity. The research presents a uniquely designed magnesium-hyaluronic acid (Mg-HA) micromotor that regulates the reaction between magnesium and body fluids via an asymmetric coating structure composed of poly(lactic-co-glycolic acid) (PLGA) and HA hydrogel [85]. This interaction continuously generates small-sized hydrogen (H₂) bubbles, thereby enhancing ultrasonic imaging by leveraging the contrast in acoustic impedance between the bubbles and surrounding body fluids. In vivo experiments revealed that post intra-articular injection, the H₂ bubbles created enhanced echo foci under ultrasound, and the distribution and movement trajectory of the micromotor—displayed in real time—showed linear or spiral motion at a speed of 40 to 45 μm/s. This novel method integrates the propulsion and imaging functionalities of H₂ bubbles. It enables real-time, noninvasive ultrasound monitoring of precise micromotor positioning within joints, thereby overcoming the challenges of low solubility and short retention time associated with traditional hydrogen therapy.

**Fig. 6.**
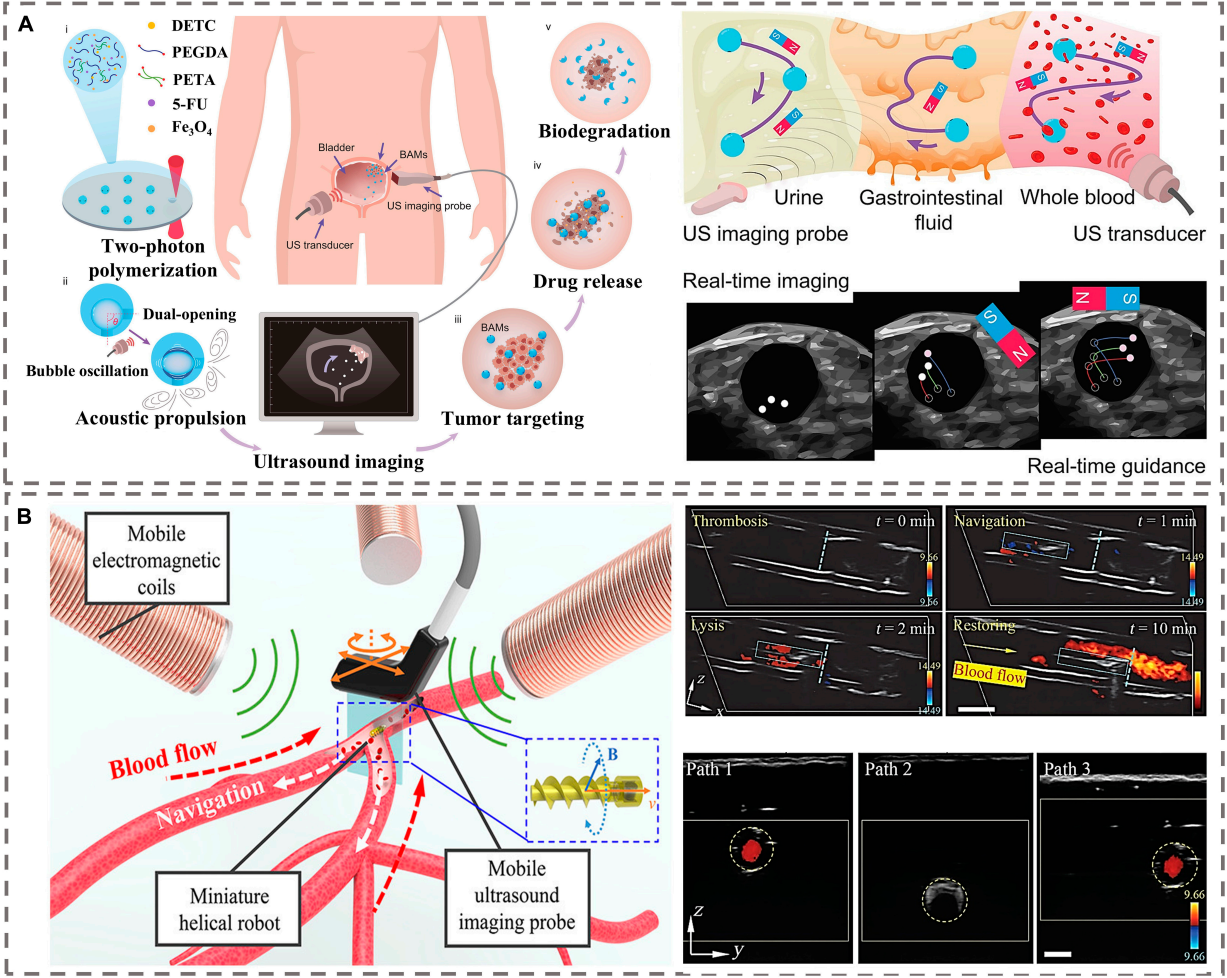
Schematic of ultrasound imaging based on MNMs. (A) The preparation and in vivo bioimaging applications of bioresorbable acoustic hydrogel microrobots. Reproduced from Ref. [86], Copyright 2024 AAAS. (B) Schematic of the ultrasound Doppler imaging-guided autonomous navigation of a miniature helical robot in a vascular environment. Reproduced from Ref. [90], Copyright 2022 American Chemical Society.

Although microbubble contrast imaging agents can substantially improve the signal of USI, their poor stability and short service life limit their use in in vivo imaging applications. These types of micromotors require specific catalytic reaction environments (e.g., stomach) and are difficult to control in terms of reaction rate and time. For long-term stability, Han et al. [86] developed a bioabsorbable acoustic hydrogel microrobot by refining its structural design, as shown in Fig. 6A. This design incorporates a double-opening bubble capture cavity, enhancing imaging efficiency. The internal spherical cavity, measuring 18 μm in diameter, can steadily capture microbubbles and induce their resonant oscillations within the ultrasonic field. Hydrophobic inner layers prolong microbubble retention, while hydrophilic outer layers prevent aggregation, enabling 14-day stability in biological fluids. These designs address the instability of conventional bubble-based agents, showcasing MNMs’ potential in precision theranostics via US-guided targeting and real-time monitoring.

#### Doppler imaging enabled by MNMs

Doppler imaging is an alternative technology for imaging MNMs with ultrasound. Doppler imaging modes with variable sensitivity and rapid imaging speeds are suitable for tracking robots in dynamic situations. Depending on the Doppler effect, 2 pulses with different pulse repetition frequencies will interact with the MNMs, resulting in different traveling frequencies for the 2 echoes. Finally, the motion behavior of motors is visualized by extracting the Doppler signal. The Doppler signal, in contrast to B-mode imaging, shows not only the MNMs but also the region that the MNMs influence. Singh et al. [87] used color Doppler to observe the velocity of MNMs; they designed hair micromotors with the potential to operate as ultrasonic contrast agents using biocompatible hair as a substrate. Loading superparamagnetic iron oxide nanoparticles into the hair micromotor allowed the micromotor to be guided in a magnetic field in response to magnetic field gradients. The hollow medullary cavity in the hair allowed the hair micromotor to be used as an ultrasonic contrast agent. Moving hair robots were visualized as dots in color Doppler mode, with the color displayed depending on their speed of movement. Importantly, the color of the markers in the Doppler mode separated the hair micromotor from the background tissue. Yan et al. [88] used ultrafast ultrasound for real-time imaging of the hydrogel micromotors. Within 2,000 frames, 551 tracks were recorded after localization and tracking. Power Doppler scans of the micromotors revealed that their movement speed is between 50 and 200 mm/s. An average speed of about 113.7 mm/s was obtained by averaging the speeds of all trajectories. This work proposed an innovative approach for real-time imaging and tracking of MNMs in deep tissues, paving the path for USI-guided MNMs in vivo. To enable imaging in complex fluid environments, ultrasound Doppler imaging is applied to guide the controlled motion of helical micromotors in complex fluids. Due to the high-frequency sound waves reflected from the red blood cells (RBCs) in the blood, ultrasound Doppler imaging is able to provide information on the velocity of the blood flow induced by the micromotors. The micromotors can perform positional tracking and recognize their surroundings more easily with the help of this type of imaging. Additionally, active targeting of nanomotors for the treatment of osteoarthritis has been effectively accomplished using USI to direct the movement of reactive oxygen scavenging nanomotors in mouse osteoarthritis [89]. MNMs such as spiral robots disrupt the surrounding blood flow and alter the movement trajectory of RBCs when they rotate, as shown in Fig. 6B [90]. This rotation of the helical structure induces a velocity gradient in the local blood flow, which, in turn, causes a Doppler frequency shift of RBCs under the high-frequency sound waves emitted by the ultrasonic probe. The ultrasonic system captures this frequency shift signal, subsequently forming a specific color area on the image. For instance, red and blue colors respectively indicate the blood flow toward or away from the probe, thereby directly signaling the position of the robot. This method addresses the issues of low signal-to-noise ratio and the challenge of identifying small-scale objects often encountered in traditional B-type ultrasound.

#### Swarming intelligence-mediated imaging enhancement based on MNMs

**Fig. 7.**
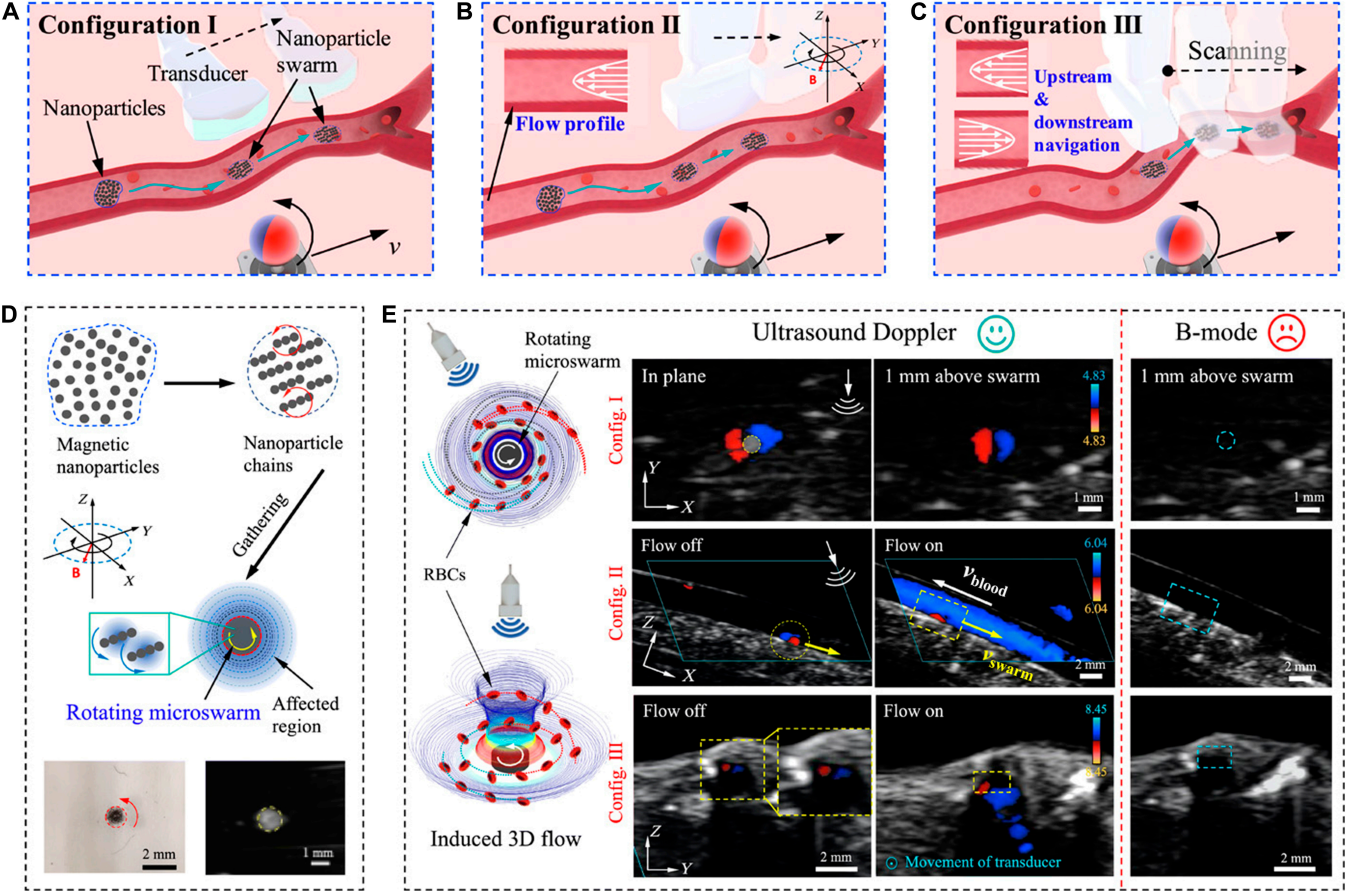
Schematic illustration of ultrasound Doppler imaging-guided swarm formation and navigation in blood vessels. (A to C) Schematic of the swarm navigation in blood vessels. The microswarm is formed, navigated, and tracked in blood vessels. (D) The formation process of a microswarm. (E) The ultrasound Doppler signal around a rotating microswarm in blood. Reproduced from Ref. [97], Copyright 2021 The Authors.

Larger micromotors show great potential for enhancing USI contrast and drug-loading ability. However, due to the confined and complex physiological environment in the biological body, the application of larger micromotors is severely limited. The swarming behavior of MNMs has been gaining attention in recent years [91–94]. The collective of MNMs is thought to be a powerful technique for overcoming this obstacle. In comparison to using a single imaging contrast agent, swarming patterns of MNMs could increase the density of the construction blocks and enhance image contrast, allowing for deep-tissue imaging [95]. The interaction between components can produce varied dynamic patterns when stimulated by external energy, allowing imaging to be realized. Using Fe_3_O_4_@polydopamine MNPs as a building block, Wang et al. [96] prepared a magnetically powered and guided microswarm. They showed that the microswarm can follow a path in real time, with an average steady-state inaccuracy of 0.27 mm (33.7% of the body length), paving the way for the in vivo implementation of microswarm guided by USI. With the ongoing improvement of MNMs technology, Wang et al. [97] demonstrated the use of Doppler USI for real-time imaging and control of magnetic MNMs in vivo, as shown in Fig. 7. Swarm formation and navigation were investigated in porcine coronary arteries by integrating a magnetic control and dynamic Doppler feedback platform. The rapid Doppler feedback allowed for real-time tracking, navigation, and localized delivery in a variety of flow conditions, and swarm-induced Doppler signals were observed at a mean velocity of up to 50.24 mm/s. This study offered a viable method for observing medication distribution in dynamic blood vascular systems and confirmed the efficacy of this delivery system in a flow environment. For the medical application of MNMs in organisms, the integration of MNMs with USI is critical. The features of USI allow for the accurate, real-time, harmless localization, and tracking of MNMs deep within tissues, particularly the blood vascular system. However, when tracking motors on the nanoscale, the resolution of ultrasonic imaging may need to be enhanced further. Because of gas and bone obstruction, USI, for example, still has serious drawbacks in terms of localization mistakes and background signals. Furthermore, while using MNMs as multifunctional contrast agents for USI is a promising future direction, enabling MNMs to improve bioimaging while performing specialized transport therapeutic functions remains a challenge.

### Magnetic resonance imaging

MRI offers multiparameter, radiation-free, high-contrast imaging for MNMs, enabled by tunable magnetic field pulses to detect diverse materials [98]. The operational principle is based on nuclear spin relaxation dynamics, wherein protons aligned in a static magnetic field undergo resonant excitation through radiofrequency (RF) pulse irradiation, subsequently emitting detectable electromagnetic signals during their coherent relaxation to thermal equilibrium state [99]. The nuclear spins will resonantly oscillate at a particular frequency when the imaging target is put in a constant static magnetic field. The imaging target is activated by an RF pulse at the proper resonant frequency, changing the net magnetization once it achieves this equilibrium magnetization. The electromagnetic signal is sent back to the spectrometer once the RF pulse ends and the spin returns to equilibrium. The generated change in the electromagnetic signal is used to create a 3-dimensional image of the imaging object when there is a linear field gradient present [100]. The inherent capacity of MRI for noninvasive interrogation of anatomical microenvironments via differential T1-/T2-weighted contrast mechanisms facilitates multiparametric tissue characterization, particularly when synergized with paramagnetically/diamagnetically engineered MNMs. Contemporary innovations exploiting spin–spin (T2) and spin–lattice (T1) relaxation dynamics have established quantitative spatiotemporal mapping paradigms for autonomous motor propulsion and biodistribution analysis.

#### T1-weighted contrast enhancement enabled by MNMs

**Fig. 8.**
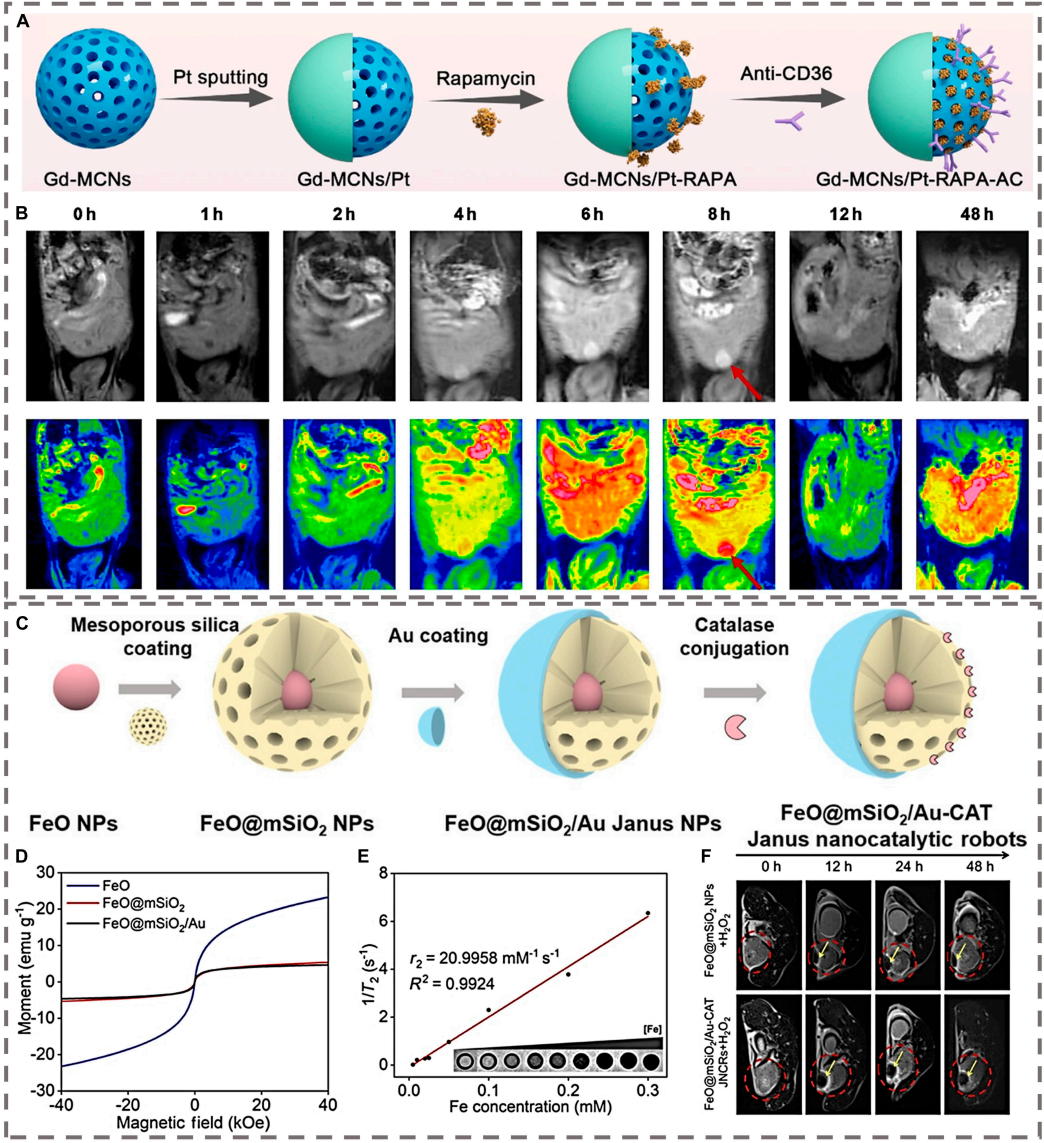
Schematic of MRI based on MNMs. (A) Schematic illustration of synthesis of Gd-MCNs/Pt-RAPA-AC nanomotors for T1-weighted MRI. (B) T1-weighted and T1 map MR images of the hepatobiliary system in mice after injection of Gd-MCNs/Pt-RAPA-AC. (C) Schematic illustration of the synthetic and fabricated procedures of FeO@mSiO_2_/Au-CAT JNCRs for T_2_-weighted MRI. (D) Magnetization curves of FeO@mSiO_2_/Au-CAT JNCRs. (E)Transverse relaxivity measurement and iron concentration-dependent T2-weighted MRI images of FeO@mSiO_2_/Au-CAT JNCRs. (F) Representative in vivo T2-weighted MR images of subcutaneous 4T1 tumors in mice after injection of FeO@mSiO_2_/Au-CAT JNCRs. (A) and (B) are reproduced from Ref. [103]. (C), (D), (E), and (F) are reproduced from Ref. [108], Copyright 2023 American Chemical Society.

Clinically, T1 MRI contrast is primarily achieved using gadolinium (Gd^3+^)-based agents, leveraging their 7 unpaired electrons to accelerate proton spin–lattice relaxation (T1) [101]. Recent advancements integrate Gd^3+^ into MNM architectures to enhance contrast efficiency and targeting. For example, Zheng et al. [102] designed NIR-driven Janus nanomotors with Gd (III)-doped mesoporous silica cores and gold hemispheres. The high surface area of these motors enabled dense Gd^3+^ doping, yielding an *r*_1_ relaxivity of 8.92 mM^−1^·s^−1^—twice that of clinical Gd-DTPA (4.37 mM^−1^·s^−1^). Under NIR irradiation, the Janus structure facilitated directional motion, improving tumor penetration and T1-weighted signal-to-noise ratio for in vivo imaging. Similarly, Gd-doped mesoporous carbon nanomotors (Gd-MCNs/Pt-RapA-AC) functionalized with anti-CD36 antibodies demonstrated enhanced atherosclerotic plaque targeting, as shown in Fig. 8A [103]. The paramagnetic Gd^3+^ shortened T1 relaxation (*r*_1_ = 27.7 mM^−1^·s^−1^). The anti-CD36 antibody, modified onto the nanoparticle surface via covalent bonds, binds specifically to the CD36 receptor on the surface of inflammatory macrophages. This binding enhances the concentration of nanomotors in atherosclerotic plaques, reduces their nonspecific distribution, and improves the imaging signal-to-noise ratio. Furthermore, the autonomous movement of the nanomotor, driven by bubble propulsion and a dual mechanism of light and heat, can penetrate the dense structure of atherosclerotic plaques and narrow endothelial cell spaces. This penetration substantially enhances deep-tissue imaging capabilities, as shown in Fig. 8B. This nanomotor addresses the limitations of traditional nanomaterials in atherosclerosis imaging, namely, their inadequate penetration and suboptimal targeting, thereby facilitating efficient MRI.

Nonetheless, the biotoxicity of the element gadolinium, which may harm the cells or tissues, is the main restriction on the use of Gd nanomotors for in-body applications in MRI. Furthermore, Gd chelates run the risk of being quickly eliminated by the kidneys, which could render them harmful for patients with impaired renal function. To mitigate Gd^3+^ biotoxicity, Mn^2+^-based alternatives have emerged. Lin et al. [104] developed Au-MnO Janus nanomotors that release Mn^2+^, a T1-shortening agent, achieving a 2.6-fold signal-to-noise ratio enhancement in hepatic tumors. The nanomotor demonstrates biocompatibility and exhibits promising potential for clinical bioimaging applications.

#### T2-weighted contrast enhancement enabled by MNMs

T2 contrast relies on superparamagnetic iron oxide nanoparticles, which induce spin–spin relaxation (T2) through magnetic dipole–dipole interactions. Biohybrid magnetotactic bacterial MNMs, inspired by natural magnetoreception, exhibit vascular penetration and T2-weighted imaging capability. For instance, Martel et al. [105] used flagellar nanomotors conjugated to magnetite vesicles for real-time MRI-guided vascular navigation, enabling clear visualization in capillary networks. Due to the ability of the nanomotors, they were able to penetrate capillaries, resulting in clear images of the nanomotors in the vascular system. Further, Martel et al. [106] developed a medical intervention platform based on magnetic–bacterial hybrid micromotors. With this platform, MRI can track and image magnetic hybrid MNMs, and the imaging information can be used to guide these synthetic MNMs in real time to perform tasks such as directional transport along preplanned vascular pathways.

Synthetic magnetic MNMs further extend T2 contrast applications. Yan et al. [70] coated *Spirulina* microalgae with superparamagnetic magnetite, achieving enhanced T2 contrast at 0.5- to 1.0-cm tissue depths. Hou et al. [107] developed erythrocyte-mimicking motors with Fe₃O₄ cores, demonstrating high *r*_2_ relaxivity (178.79 mM^−1^·s^−1^) and biocompatible clearance. The nanomotor was constructed from natural maize alcohol-soluble proteins and disguised with RBC membranes, and functional cargoes such as Fe_3_O_4_ nanoparticles and the chemotherapeutic drug adriamycin were loaded into the nanomotor. The nanomotor could be accurately guided by an externally rotated homogeneous magnetic field and applied as an MRI contrast agent for tumor imaging because of the massive loading of Fe_3_O_4_ nanoparticles. These motors, guided by external magnetic fields, exhibited concentration-dependent T2 signal enhancement and minimized nonspecific accumulation. To address the challenge of tracking MNMs in real-time imaging within tumors, FeO@mSiO_2_ nanoparticles were used to construct FeO@mSiO_2_/Au-CAT Janus nanomotors, as shown in Fig. 8C [108]. FeO@mSiO_2_ can be used for T2-weighted MRI because of their superparamagnetic nature. Self-propulsion of the nanomotors achieved deeper tumor tissue penetration. Higher T2 signals were observed in deeper tumor tissues following peritumoral injection of FeO@mSiO_2_/Au-CAT Janus nanomotors, as opposed to passive FeO@mSiO_2_ nanoparticles, indicating that JNCRs mediated deeper tumor penetration. This work demonstrated the potential of MNMs for imaging-guided tumor therapy by successfully performing MRI of MNMs in deep tumors.

Dual-modal T1/T2 contrast is achieved in NiFe₂O₄-loaded MoS₂ MNMs (MNBOTs), where paramagnetic Ni^2+^/Fe^3+^ ions shorten both T1 and T2 relaxation [109]. The inherent paramagnetic properties of NiFe₂O₄ allow it to exhibit high signal intensity in T1-weighted imaging (shortening T1), while the magnetic dipole–dipole interactions during particle aggregation intensify T2 relaxation (shortening T2), facilitating dual-mode contrast. In T2-weighted imaging, the signal intensity for the 0.2 mg/ml MNBOTs group was over 3 times greater than that of the control group, with a clear signal boundary and minimal diffusion artifacts, underscoring the enhancement in resolution via magnetic navigation and targeted enrichment.

While MNMs offer precise spatial targeting and contrast enhancement, clinical translation faces hurdles. Real-time MRI tracking demands faster imaging protocols to balance resolution and speed, and biocompatible design is essential for safe in vivo applications. Multimodal integration, combining MRI with USI or fluorescence, holds promise for addressing these challenges, enabling synergistic enhancement of imaging resolution and navigation accuracy.

### Computed tomography

Computed tomography (CT) generates cross-sectional anatomical images by reconstructing x-ray attenuation profiles through tissue layers [110]. X-rays transmitted through the body are converted to electrical signals via detectors, processed into digital data, and reconstructed into axial, coronal, or sagittal planes. While CT offers high spatial resolution and tissue penetration, its limited soft-tissue contrast necessitates exogenous agents to amplify x-ray absorption differences.

**Fig. 9.**
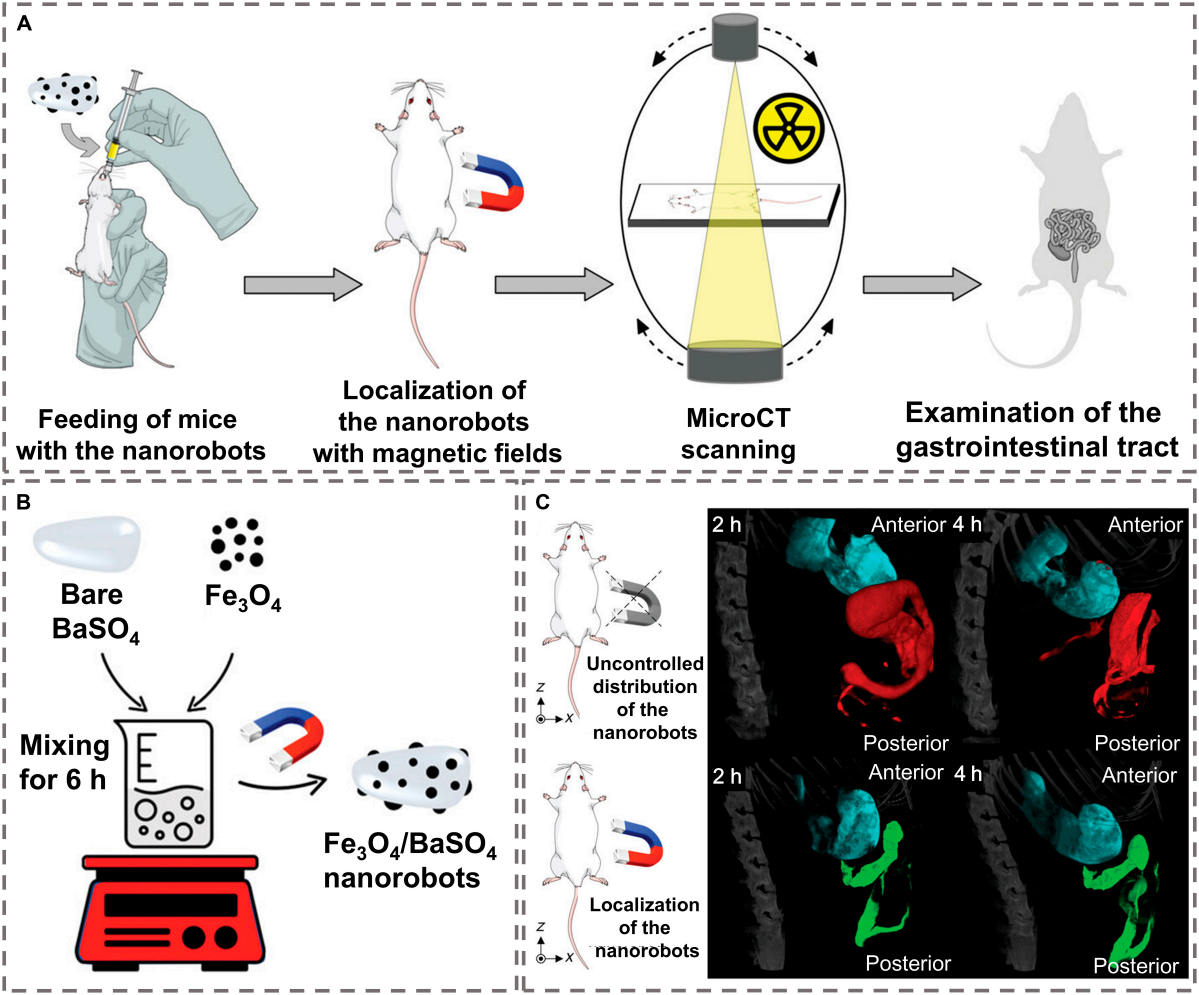
Schematic illustration of MNMs for CT imaging. (A) Localized imaging of the gastrointestinal (GI) tract via magnetically navigable Fe_3_O_4_/BaSO_4_ nanorobots. (B) The preparation of Fe_3_O_4_/BaSO_4_ nanorobots. (C) In vivo microcomputed tomography scanning of mice via Fe_3_O_4_/BaSO_4_ nanorobots. (A), (B), and (C) are reproduced from Ref. [111], Copyright 2022 Wiley-VCH GmbH.

MNMs address conventional contrast agent limitations (e.g., passive distribution and poor targeting) by integrating motion capabilities and high-atomic-number elements for enhanced x-ray attenuation. Combining BaSO₄ with magnetite (Fe₃O₄), researchers developed magnetically guided nanomotors capable of active localization in microfluidic channels and mouse intestines, as shown in Fig. 9 [111]. The high x-ray attenuation of BaSO₄ and superparamagnetic Fe₃O₄ enabled controlled movement (5.6 μm/s under rotating magnetic fields), allowing precise delineation of luminal structures. This overcomes the static distribution of traditional BaSO₄ suspensions, demonstrating targeted CT imaging in complex biological environments. Gold nanoparticles (AuNPs), renowned for strong x-ray absorption, were integrated into halloysite nanotubes (HNTs) to create photothermally driven nanomotors [112]. The AuNPs serve as a crucial imaging unit, and their SPR effect not only provides the nanomotor with the photothermal conversion ability of NIR light response but also establishes it as a contrast agent for CT imaging due to its robust x-ray attenuation characteristics. The nanomotor’s autonomous movement, propelled by NIR light and achieving maximum speeds of 64.5 μm/s, facilitates active migration and adhesion to tumor cells, thereby augmenting its penetration and aggregation within tumor tissues. In vivo experiments reveal that the CT signal intensity at the tumor site in mice injected with nanomotors was 9.2 times greater than that of the noninjection group. This enhancement is attributed to the high x-ray absorption characteristics of AuNPs and the active targeting movement of nanomotors. This design addresses limitations of passive agents, such as low permeability and nonspecific distribution. In an endeavor to achieve enhanced precision in imaging the tumor microenvironment, Zhang et al. [113] engineered spindle-shaped Janus nanomotors with Mn-MOF (metal-organic framework) and Pt nanoparticles (Pt NPs) for synergistic CT contrast. Mn-MOF decomposes tumor-specific GSH, releasing Mn^2+^ ions (*Z* = 25) to enhance x-ray attenuation, while Pt NPs (*Z* = 78) and FePc (iron phthalocyanine) leverage their high atomic numbers to amplify x-ray absorption. The Janus-type micro/nanoarchitecture achieves synergistic multimodal propulsion through catalytic decomposition of hydrogen peroxide and NIR light-driven photothermal conversion, whichsubstantially enhances tumor tissue penetration depth and promotes localized enrichment of contrast-active manganese ions. This innovative multimechanism integration strategy not only amplifies CT signal intensity by 2.3-fold compared to conventional agents but also enables precise theranostic integration through real-time imaging-guided photothermal ablation. While autonomous nanomotors have revolutionized targeted delivery paradigms by overcoming biological barriers through active locomotion, critical challenges persist in clinical translation, particularly regarding long-term biosafety evaluation (e.g., immune response modulation), spatiotemporal targeting accuracy under hemodynamic conditions, and signal-to-noise ratio optimization in deep tissues.

### Emission computed tomography

Emission computed tomography (ECT), encompassing single-photon emission computed tomography (SPECT) and positron emission tomography (PET), enables functional imaging by detecting radionuclide-derived gamma photons [114]. SPECT utilizes single-photon emitters (e.g., ^99m^Tc) with long half-lives, offering cost-effective metabolic imaging but limited spatial resolution (4 to 10 mm). PET, conversely, employs positron-emitting nuclides (e.g., ^18^F-FDG) that undergo annihilation to produce paired gamma photons, enabling high-resolution (1 to 2 mm) quantitative metabolic mapping, though requiring on-site cyclotron production due to short half-lives [115]. Both modalities often fuse with CT/MRI (SPECT/CT and PET/CT) to integrate functional signals with anatomical localization, critical for precision oncology and neurodegenerative disease diagnosis.

The autonomous locomotion of micro/nanoscale propulsion systems coupled with their programmable payload encapsulation capability effectively overcomes 2 critical shortcomings in ECT, insufficient tracer retention time and nonspecific biodistribution patterns, through spatiotemporally controlled delivery of radioisotopes with enhanced penetration of biological barriers. Iacovacci et al. [116] pioneered the integration of SPECT technology with MNMs. The hydrogel matrix retained 80% to 95% radioactivity for 2 h, mitigating rapid tracer washout and improving signal stability compared to free radiopharmaceuticals. Under external magnetic fields, these motors navigated microfluidic channels, demonstrating active localization in myocardial ischemia models—an approach that compensates for SPECT’s inherent low resolution by enhancing target-to-background ratios through controlled accumulation.

For PET, short-lived positron emitters (e.g., ^18^F and ^124^I) are integrated into nanomotor platforms to breach biological barriers. Gold-coated Au/PEDOT/Pt micromotors, surface-labeled with^124^I, exhibited high radiolabeling efficiency (95%) and stability, enabling PET-CT imaging of deep-tissue targets [117]. These micromotors possess a gold surface that is radioactively labeled by chemically adsorbing the iodine isotope ^124^I, presenting both high labeling efficiency and robust stability. The high-energy γ-photons emitted by ^124^I enable deep-tissue penetration through opaque biological matrices, while the platinum-catalyzed H₂O₂ decomposition propulsion system of nanomotors potentiates interstitial transport in tumor microenvironments, resulting in 30% elevation of tumor-specific ^18^F-FDG accumulation compared with diffusion-limited passive carriers.

**Fig. 10.**
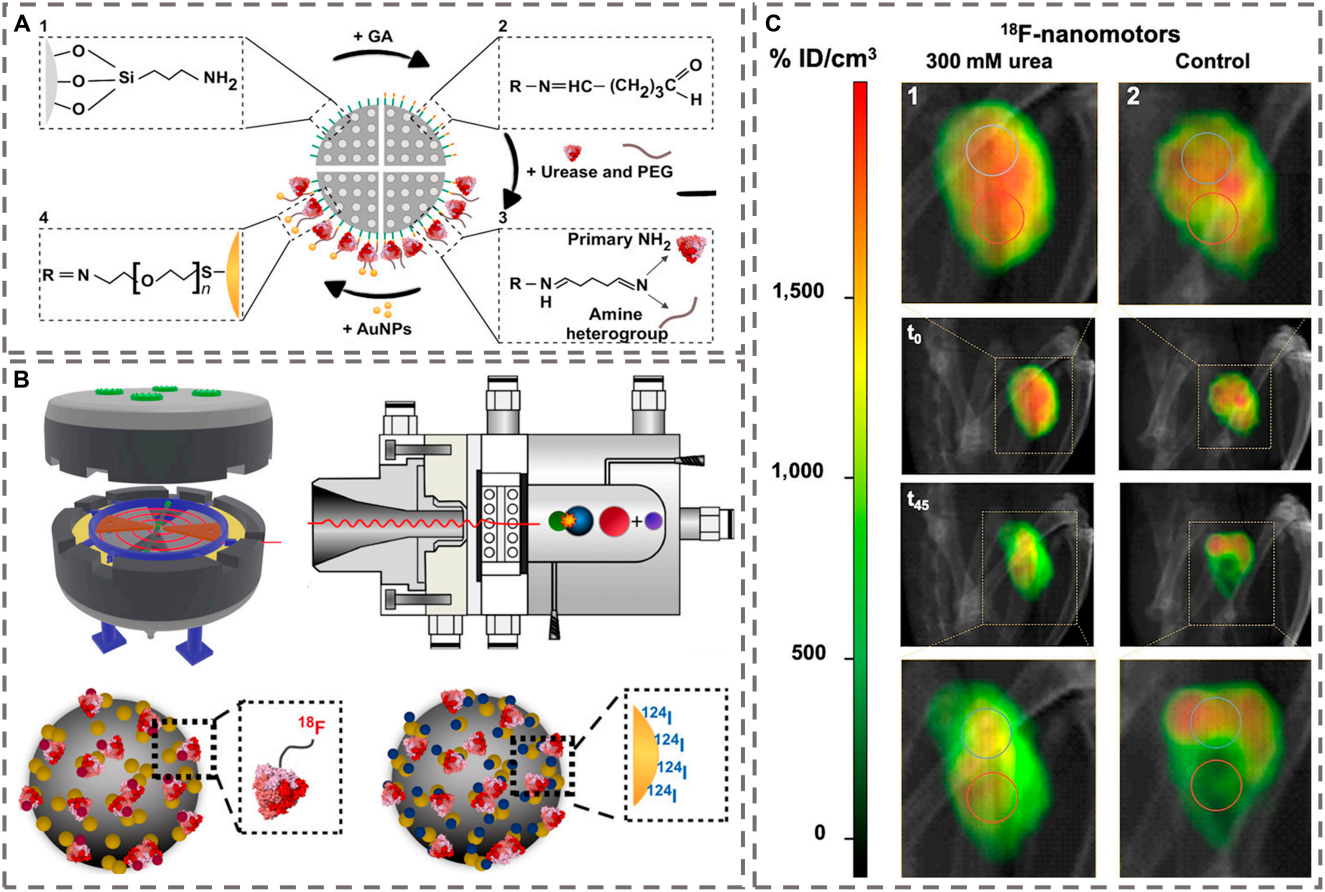
Schematic illustration of MNMs for ECT imaging. (A) Preparation of urease-powered, AuNP-decorated nanomotors. (B) Nanomotors radiolabeling using either ^18^F or ^124^I to yield ^18^F-nanomotors or ^124^I-nanomotors, respectively. (C) PET-CT images obtained at different time points after the intravesical instillation of ^18^F-labeled urease nanomotors. (A), (B), and (C) are reproduced from Ref. [118], Copyright 2021 AAAS.

Notwithstanding the inherently constrained imaging performance of single-agent probes, emerging strategies employing MNM collectives with swarm intelligence-enhanced targeting demonstrate improved spatial uniformity in contrast signal distribution—a critical advancement enabled by synergistic signal amplification mechanisms derived from coordinated population dynamics. Mesoporous silica-based swarm micromotors, functionalized with urease and AuNPs, demonstrated collective behavior to improve imaging uniformity, as shown in Fig. 10 [118]. Urease enabled urea-driven self-propulsion, while covalently bound ^18^F- and ^124^I-labeled AuNPs provided dual-tracer capability. In bladder cancer models, intravesical injection of these swarms resulted in 2.5-fold higher tracer retention than static particles, facilitated by collective fluid mixing that homogenized distribution and reduced background noise. This strategy addresses challenges in hollow-organ imaging, where passive agents often exhibit uneven accumulation.

While MNMs show promise in active ECT contrast, clinical translation hinges on resolving. These include overcoming issues related to material toxicity, motion control, and equipment compatibility. Future advancements in this field will require the integration of innovative materials, intelligent drive mechanisms of MNMs, and multimodal imaging technology.

### Photoacoustic imaging

Photoacoustic imaging (PAI), which amalgamates the superior ultrasonic penetration depth and the high spatial resolution of light, currently stands as the most promising method in bioimaging [119,120]. When short-pulse lasers irradiate tissues, endogenous chromophores (e.g., hemoglobin and melanin) absorb light energy, converting it into heat that induces thermoelastic expansion and generates ultrasonic waves. These waves are detected by external transducers and reconstructed into maps of optical absorption, enabling visualization of physiological parameters like blood oxygenation, metabolism, and vascular architecture. Despite its noninvasive advantages, PAI faces challenges: limited endogenous contrast restricts tumor specificity, while light attenuation and scattering degrade signal-to-noise ratio in deep tissues [121]. MNMs address these limitations by serving as active photoacoustic contrast agents with enhanced targeting and signal amplification.

#### Light-absorbing MNMs for signal enhancement

**Fig. 11.**
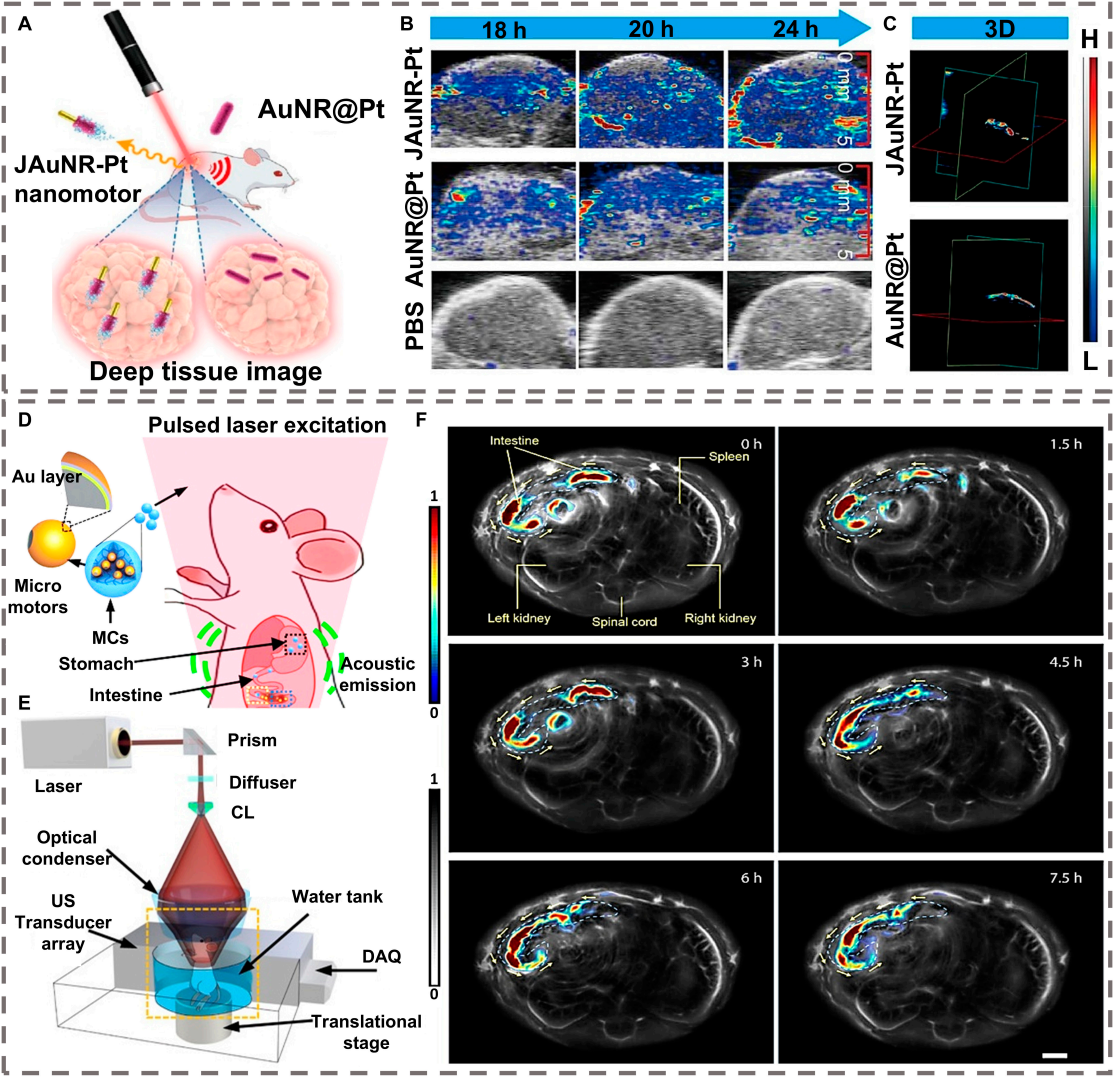
(A) Illustration of the permeability of nanomotor for deep-tissue PA imaging. (B) PA images of tumor in vivo after injection of the JAuNR-Pt nanomotor. (C) 3D PA images of tumor in vivo. (D) Schematic of the PAMR in the GI tract. (E) Schematic of PACT of the MCs in the GI tract in vivo. (F) Time-lapse PACT images of the MCs in intestines for 7.5 h. Scale bar, 2 mm. (A), (B), and (C) are reproduced from Ref. [125], Copyright 2022 American Chemical Society. (D), (E), and (F) are reproduced from Ref. [127], Copyright 2019 The Authors.

MNM designs leverage plasmonic or photothermal materials to amplify PA signals while enabling directional motion for targeted delivery. To boost NIR absorption, iron (III) oxide nanoparticles were embedded in *Spirulina* microalgae, followed by polydopamine (PDA) coating to form PDA-magnetized *Spirulina* (MSP) micromotors [122]. The aromatic PDA layer exhibited strong 808-nm absorption, converting light energy into localized heat via the photothermal effect, which enhanced thermoelastic expansion and PA signal intensity. By optimizing PDA thickness, the PA signal of PDA-MSP showed a concentration-dependent increase, surpassing uncoated controls by 30%. Magnetic guidance allowed precise localization in microfluidic channels, demonstrating potential for navigating complex biological environments. To overcome the challenges associated with limited accumulation and uneven distribution of contrast agents in tumors, Liu et al. [123] developed Au-TiO₂ heterojunction nanomotors with asymmetric structures to enhance local surface plasmon resonance (LSPR) in the NIR-II window (1,000 to 1,700 nm). The Au nanorods (Au NRs) exhibited a red-shifted LSPR peak to 1,050 nm after TiO₂ modification, increasing light absorption efficiency for deep-tissue penetration. Under NIR illumination, plasmonic enhancement drove photocatalytic H₂ bubble generation, propelling the nanomotors at 5.06 μm/s to overcome tumor interstitial pressure. This active movement improved tumor accumulation by 1.8-fold compared to passive nanoparticles, with PA signal intensity 2.5 times higher than pure Au NRs. In a similar pursuit to enhance PAI, Aziz et al. altered the surface of magneto-driven Janus micromotors by incorporating gold nanorods (AuNRs) and gold nanofilms (AuNSs), both of which exhibit pronounced light absorption capabilities. This enhancement in the photoacoustic signal is achieved by leveraging the LSPR effect. By meticulously adjusting the distance and structure between the nanomaterial and the micromotor substrate, they optimized the light absorption characteristics. This optimization ensures the absence of interference from the substrate metal, thereby preserving its optical properties and yielding highly specific signals for deep-tissue imaging [124]. The impact of contrast agent size on PAI effectiveness is pronounced. Consequently, nanomotors, measuring less than 100 nm, were manufactured utilizing gold nanorod-platinum. These motors are capable of producing robust NIL-II PA imaging signals, making them suitable for deep in vivo imaging applications, as shown in Fig. 11A [125]. Specifically, the release of platinum ions expedited the onset of DNA damage and apoptosis. When exposed to hydrogen peroxide, the nanomotor demonstrated swift autonomous movements, thereby augmenting cellular uptake and hastening lysosomal escape. Leveraging these advantages, this study successfully employed the nanomotor for in vivo tumor imaging, offering a novel approach to the amalgamation of tumor diagnosis and treatment.

For tumor imaging, MNM-enhanced PAI demonstrates considerable promise in thrombus imaging. Su et al. [126] employed silica nanospheres as templates to sequentially construct gold shells and platinum layers, thereby fabricating bowl-shaped nanomotors. The nanomotor surface was modified with urokinase (UK) and specific fibrin peptide (CREKA). This modification leveraged the high affinity of the CREKA peptide for fibrin, enabling the nanomotor to specifically target thrombosis sites. The gold shell’s strong absorption in the NIR window led to a linear increase in PA signal intensity with increasing nanomotor concentration. This provided a clear visual representation of the thrombus location and size, offering a valuable tool for precision medicine.

#### Multifunctional integration with photoacoustic tomography based on MNMs

In an effort to further refine deep PAI, the integration of MNMs with photoacoustic computed tomography (PACT) has been employed. Mg-Au micromotors were prepared for target imaging in intestines in vivo, as shown in Fig. 11D [127]. The Au layer is utilized to improve the deep-tissue imaging capabilities of the micromotors for clinical applications by increasing the optical absorbance for PA imaging and effective propulsive response rate at the same time. By detecting photon-induced ultrasonic waves, PACT imaging offers high-resolution imaging at depths much beyond the optical diffusion limit. PACT delivers extraordinarily high-depth spatial resolution, deep penetration, and anatomical and molecular contrast at fast imaging rates by making use of the soft tissue’s little acoustic dispersion. PACT-guided MNMs are anticipated to be included in future in vivo multifunctional bioimaging and treatment platforms.

MNMs have demonstrated substantial potential in enhancing the capabilities of single-modal imaging techniques, as summarized in Table 1. While their active motion improves target specificity and signal contrast in individual modalities, single-modal imaging inherently faces limitations rooted in its one-dimensional information capture. Most single-modal techniques either prioritize structural visualization (e.g., CT and USI) or functional mapping (e.g., PET and PAI), but rarely achieve concurrent high-precision depiction of both. In clinical practice, patient pathologies often manifest as complex, multifactorial conditions requiring comprehensive diagnostic information. The resultant lack of integrated structural-functional data hinders systematic evaluation of disease states, potentially compromising the precision of clinical decision-making. Addressing these limitations, the evolution toward multimodal imaging represents an essential paradigm shift in modern diagnostics. By fusing complementary modalities, clinicians can acquire layered information that bridges anatomical context with functional dynamics, enabling holistic disease characterization. The following chapter systematically investigates MNM architectures integrating multimodal imaging payloads, facilitating seamless integration of active targeting with multidimensional signal acquisition.

**Table 1.** Summary of single-modal bioimaging for MNMs

Mode	Enhancement paradigms	Materials	Advantages	Ref.
FI	Fluorescent functionalization	ABF-NIR-797	Deep-tissue penetration	[57]
		FLA/silica-NH_2_/Pt	High sensitivity, low cost, operational simplicity	[54]
		ABFs-liposomes-calcein	High sensitivity, low cost	[55]
		JN@MnO_2_@hQN	Intracellular imaging	[58]
		FAM-Cy5-Au-SiO_2_-NH_2_	Intracellular imaging	[59]
		PS@PDA-ICG	Deep-tissue penetration	[60]
	Spectral engineering	CdTe QDs-PEDOT-Pt	High luminescence efficiency	[63]
		CdTe QDs-DOX-Fe_3_O_4_	Excellent photostability	[64]
		PABA- GQDs	Excellent photostability, biocompatibility	[67]
		Cu_2_O@PbS	Deep-tissue penetration	[68]
	Bioluminescence	*S. platensis*-Fe_3_O_4_	Deep-tissue penetration, biocompatibility	[70]
		*Spirulina platensis*-Fe_3_O_4_	Biocompatibility	[71]
		Volvox-Fe_3_O_4_	Biocompatibility	[72]
	AIE	PEG @PTMC	High-quality signals	[75]
		Pt-MOFs@TPPE	High-quality signals	[76]
UI	Microbubble	Platinum-PEDOT-catalase	Noninvasive, fast, safe, low-cost, deep penetration	[82]
		Mg/Ni	Noninvasive, biocompatibility	[83]
		Hollow microtubes	Noninvasive, biocompatibility	[84]
		Mg-HA-PLGA	High contrast	[85]
		Hydrogel	High contrast, biocompatibility	[86]
	Doppler	PEGDA-NdFeB	High temporal resolution	[90]
		Hair-Fe_3_O_4_	Biocompatibility, low cost	[87]
		Hydrogel	High contrast, biocompatibility	[88]
		Prussian blue-Fe_3_O_4_	Noninvasive, low cost	[89]
	Swarm	Fe_3_O_4_@PDA-NPs	High temporal resolution, high signal-to-noise ratio	[96]
		Fe_3_O_4_	High temporal resolution, high signal-to-noise ratio	[97]
MRI	T1-weighted	Gd (III)-Au-SiO_2_	High tissue contrast, high temporal resolution	[102]
		Gd-MCNs/Pt-RapA-AC/anti-CD36	High tissue contrast, high temporal resolution	[103]
		Au-MnO	High tissue contrast, deep penetration	[104]
	T2-weighted	FeO@mSiO_2_/Au-CAT	High tissue contrast, high temporal resolution	[108]
		Flagellated magnetotactic bacteria	High tissue contrast, high temporal resolution	[105]
		protein zein- Fe_3_O_4_-DOX	High tissue contrast, high temporal resolution	[107]
	T1/T2-weighted	NiFe_2_O_4_-loaded MoS_2_	High temporal resolution	[109]
CT		Fe_3_O_4_/BaSO_4_	Fast, 3-dimensional	[111]
		Au@HNTs	Fast, 3-dimensional	[112]
		Pt/FePc@Mn-MOF	Fast, 3-dimensional	[113]
ECT	SPECT	^99m^Tc [Tc], NIPAAM, PEGDA	Low-radiation, high sensitivity	[116]
	PET-CT	Au/PEDOT/Pt	High sensitivity, fast imaging	[117]
	PET	^124^I, ^18^F, urease, AuNPs	High sensitivity, fast imaging	[118]
PAI		Magnetized PDA-MSP	Deep penetration, high contrast	[122]
		Au-TiO_2_	High contrast, deep-tissue penetration and sensitivity	[123]
		AuNRs@AuNSs	High contrast, deep-tissue penetration and sensitivity	[124]
		AuNR@Pt	High spatial resolution, deep-tissue penetration	[125]
		UK@CREKA peptides	High spatial resolution, deep-tissue penetration	[126]
	PACT	Mg-Au	High spatiotemporal resolution, deep penetration	[127]

## Innovations in Multimodal Integration Based on MNMs

MNMs substantially enhance multimodal fusion imaging by capitalizing on the strengths of various imaging modalities. These strengths include the deep-tissue penetration of MRI, the molecular specificity of FLI, and the high resolution offered by PAI. The enhancement is achieved through the development of multifunctional carriers, the synchronization of multifield responses, and the integration of cross-modal signals. This strategy effectively surmounts the limitations inherent in individual imaging modalities. In terms of material design, MNMs have the unique capacity to simultaneously load a variety of probes such as fluorescence, magnetism, and photoacoustic. This allows them to generate multimodal signals triggered by a single carrier. Furthermore, regarding the driving mode, an external field, such as light, magnetism, and ultrasound, serves dual functions. It not only propels the movement of the MNM but also acts as an excitation source for the imaging mode. Consequently, this creates a natural system where driving and imaging collaborate seamlessly.

### Integrated design of MNMs as imaging agents

Different modal probes are integrated into the MNMs system through core–shell or composite structures to achieve synchronous excitation and complementary output of signals. Chen et al. [128] developed a dual-source dynamic Janus nanomotor that integrates multimodal imaging using a “core–shell–Janus” structural design. This motor establishes a core–shell structure by coating upconversion nanoparticles (UCNPs) with mesopore silica on one side of the gold shell. The UCNPs’ NIR light is converted into visible blue light, which drives the FLI of photosensitizers and photodynamic therapy. The gold shell facilitates PAI and surface-enhanced Raman scattering (SERS) detection, quantifying H₂O₂ using a 3-MPBA probe. Concurrently, the surface-modified catalase aids in the production of H₂O₂ oxygen, complementing the photothermal effect of the gold shell to create a dual-source power. This enhances the nanomotors’ active penetration into tumor tissues and ameliorates the hypoxic microenvironment. The design enables the multimodal fusion of SERS molecule detection, FLI cell localization, and in vivo PAI imaging. Liu et al. [129] developed an MNM platform for multimodal imaging by functionally coupling UCNPs, AuNPs, and photosensitizers (Cys). The UCNPs emit a 659-nm red light under 980 nm excitation, enabling tumor FLI while also emitting an 800-nm NIR light for real-time monitoring of singlet oxygen levels, thus providing online feedback on therapeutic effects. The AuNPs serve as photothermal conversion agents, nanomotor driving sources, and catalytic enzymes. They drive autonomous movement via an asymmetric thermal gradient produced under an 808-nm laser, which increases tumor penetration depth to 100 μm. Additionally, the AuNPs decompose H₂O₂ to generate oxygen, alleviate tumor hypoxia, and enhance the photothermal/photodynamic (PTT/PDT) synergistic effect. The Cys works in tandem with the AuNPs to enhance the photothermal effect and produce reactive oxygen species. This integrated system enables upconversion luminescence imaging, PAI, and FLI. It breaches the tumor barrier through the active penetration of nanomotors. The system’s combination of AuNP microenvironment regulation and multiple component functional synergy achieves a “diagnosis–treatment–monitoring” closed loop. In vitro and in vivo experiments have confirmed its high antitumor efficacy and biocompatibility, offering a novel nano-platform for accurate diagnosis and treatment of tumors.

Covalent organic frameworks (COFs) have been employed in the development of multimodal imaging agents. In this research, Feng et al. [130] crafted a COF-based core–shell–membrane structured biomimetic nanomotor (mPPy@COF-Por). The inner polypyrrole (PPy) core facilitates directional motion via self-thermal swimming under NIR light, augmenting tumor-targeted enrichment. The external shell, modified with porphyrin-COF, possesses both photodynamic therapy and FLI capabilities. A coating derived from the HCT116 cancer cell membrane ensures homologous recognition and minimizes bioadhesion. Merging the infrared thermal imaging and PAI data from PPy, along with the FLI data from porphyrin, and enhancing active targeting and biocompatibility through light-driven motion, this nanomotor synergistically enhances infrared thermal/photoacoustic/fluorescence tri-modal imaging in both in vitro cellular contexts and in vivo tumor models. Such a platform offers a versatile, integrated approach for precise tumor diagnosis and combined photothermal and photodynamic therapies, showcasing the pioneering potential of COF-based nanomaterials in biological imaging.

**Fig. 12.**
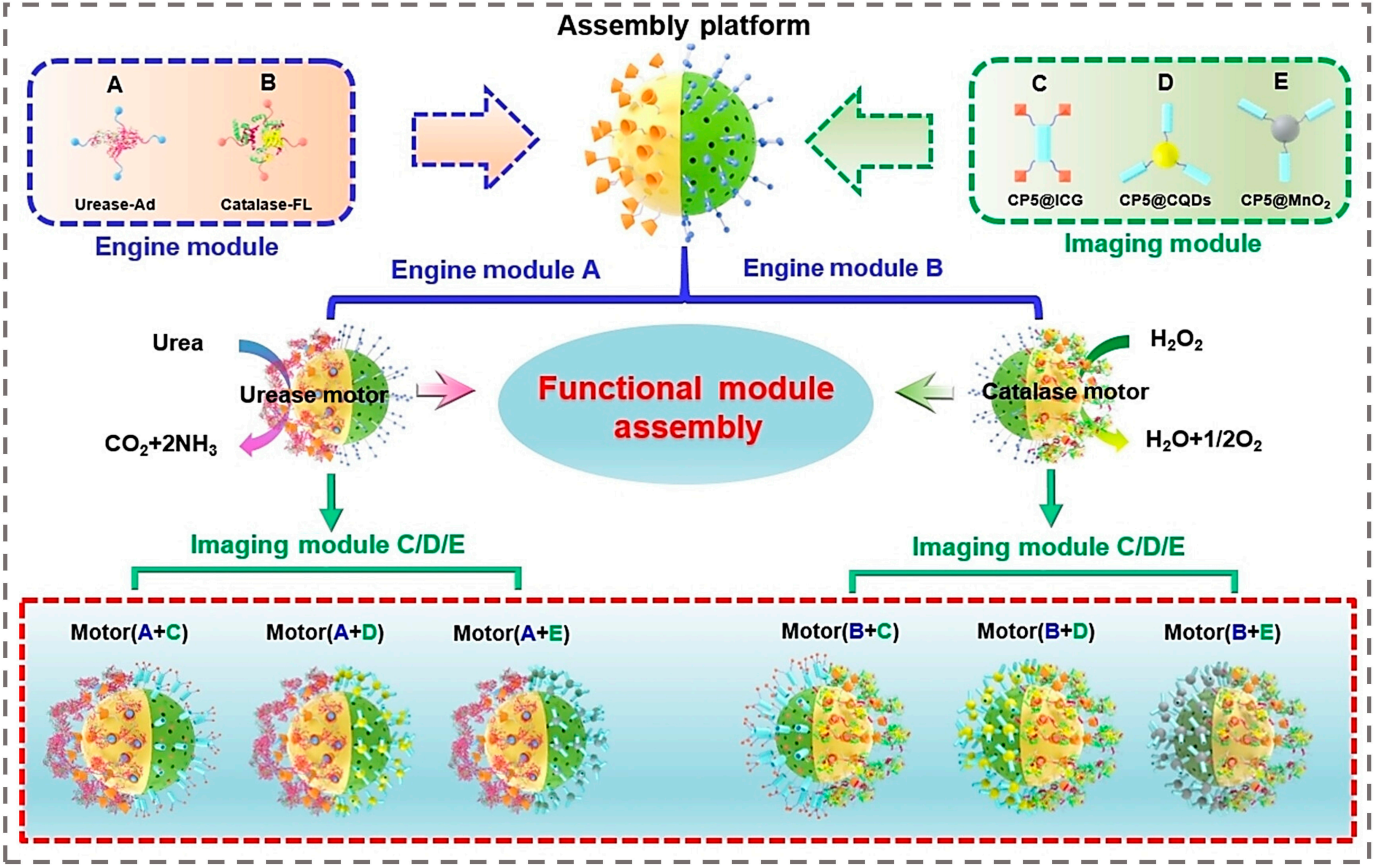
Schematic illustration of on-demand construction of imaging nanomotors using a modular assembly strategy based on host–guest interaction. Reproduced with permission [131], Copyright 2024 Wiley-VCH GmbH.

Multimodal imaging can be achieved not only by directly integrating imaging probes onto the MNM platform but also through the switchable assembly of diverse imaging modules with MNMs. This is facilitated by the modular assembly strategy of supramolecular host–guest chemistry, which enables the construction of MNMs that can integrate driving and imaging functions as required, as shown in Fig. 12B [131]. Using hollow mesoporous silica nanoparticles as a universal carrier, their Janus surface is functionalized with thiol-β-cyclodextrin (S-β-CD) and trimethoxysilylpropylethylenediamine (MC) as binding sites for enzyme-driven (urease/catalase-adamantine/fluorescein) and imaging modules (ICG, carbon quantum dots [CQDs], and BMnO₂), linked via carboxyl pillararene CP5. Reversible interactions allow modular assembly, and enzyme-catalyzed propulsion boosts nanomotor diffusion (e.g., 256% increase in urease motors at 300 mM urea). Combined with ICG’s deep penetration, CQDs’ intracellular labeling, and BMnO₂’s MRI contrast, this enables dynamic tracking in cells and mouse bladders with good biocompatibility. The “universal platform + functional modules” strategy overcomes traditional limitations, offering an efficient design for programmable multifunctional MNMs in biological imaging.

### The application of multimodal imaging based on MNMs

In the realm of biomedical imaging, monomodal technologies inherently face limitations in penetration depth, resolution, and specificity. Dual-modal imaging strategies have emerged as a pragmatic solution to mitigate these constraints, offering complementary advantages unattainable by single-modal approaches. A paradigmatic example is the synergistic integration of USI and PAI, which collectively enhance imaging quality and afford multidimensional information. US, characterized by high spatiotemporal resolution and exceptional deep-tissue penetration, excels in acquiring anatomical structural details, whereas PAI leverages tissue optical properties—such as vascular architecture and oxygen saturation—to provide high-contrast functional/molecular insights. This modal combination optimizes the trade-off between imaging depth and resolution, addressing a critical challenge in clinical diagnostics.

The integration of MNMs with US and PAI has demonstrated efficacy in monitoring dynamic drug release processes. Aziz et al. [132] reported a dual-modal micro-motor design comprising a silica core encapsulated by a titanium–iron–titanium (Ti–Fe–Ti) layered structure. The iron intermediate layer confers magnetic responsiveness for external field-guided navigation, while the titanium coatings ensure biocompatibility and mechanical stability. Critically, the metallic layers’ NIR light absorption pronouncedly amplifies PAI signals, and their acoustic impedance mismatch with surrounding tissues enhances US reflection contrast. This creates an anatomical–functional dual-modality synergy—“US for structural mapping and PAI for molecular profiling”—enabling precise tracking of DOX-loaded micromotors during in vivo delivery. By capitalizing on the complementary strengths of dual-modal imaging, this study overcomes the inherent limitations of monomodal techniques, providing a technical framework for the controlled manipulation of MNMs in complex biological environments and advancing their utility in biomedical interventions. Future research may focus on optimizing material biodegradability and enhancing deep-tissue penetration to facilitate clinical translation.

**Fig. 13.**
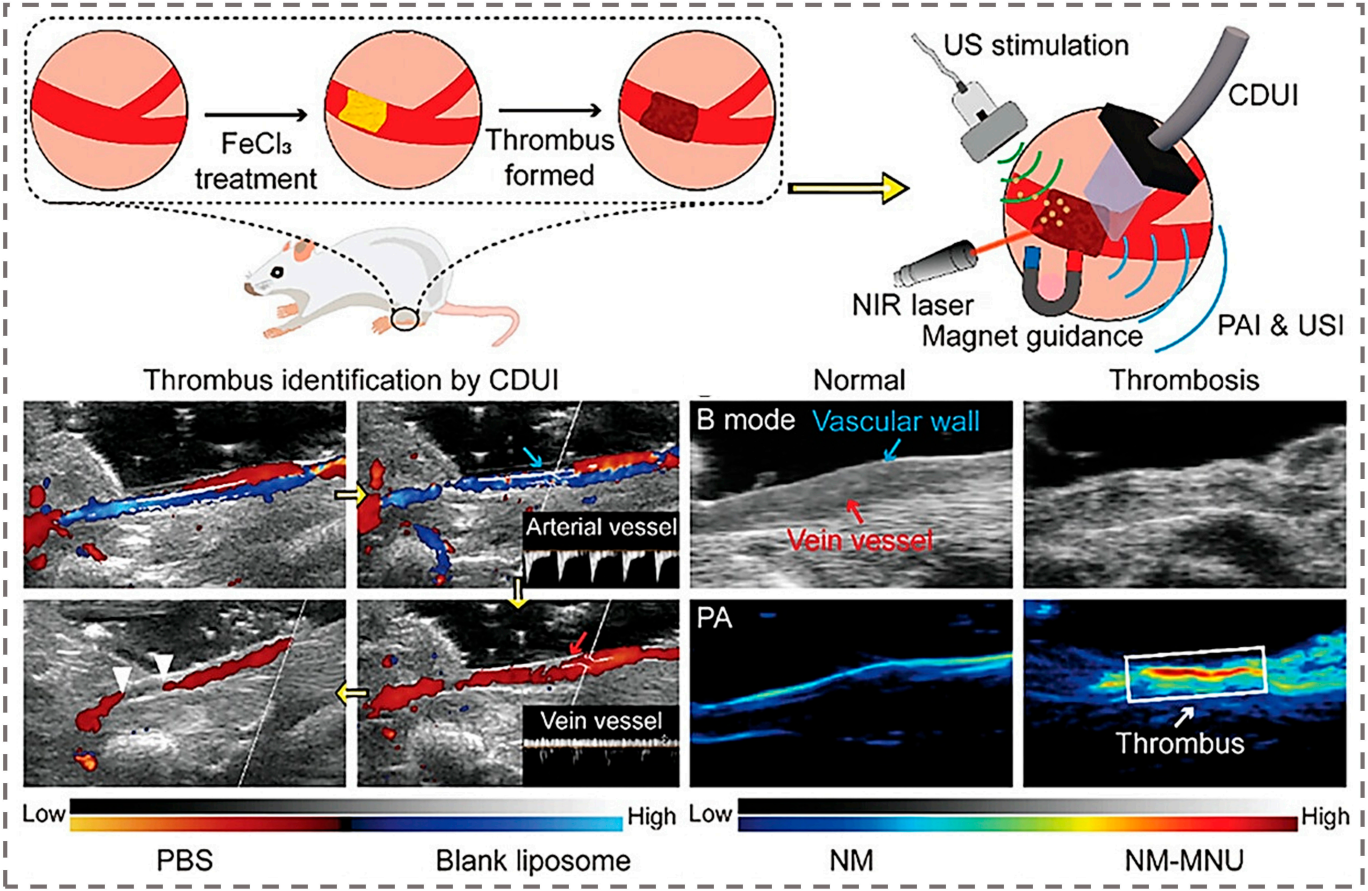
Monitoring of the thrombolytic process in vivo guided by multimodal imaging mediated by nanomotors [133], Copyright 2023 Wiley-VCH GmbH.

Multimodal imaging platforms based on MNMs have also emerged as transformative tools in thrombus imaging and theranostics. Particularly within deep thrombus, PA markedly enhances the discrimination between the target and the background, thus compensating for the deficiencies in detection of US in low signal-to-noise ratio scenarios. Nanomotors, constructed from iron oxide nanoparticles (Fe_3_O_4_), perfluorohexane (PFH), UK, and liposomes, possess a unique composition that underpins their crucial role in multimodal imaging, as shown in Fig. 13 [133]. In PAI, PFH undergoes a liquid–gas phase transition when subjected to NIR/ultrasound stimulation, thereby forming bubbles and markedly enhancing the photoacoustic signal of the nanomotors. Research indicates that, without NIR/US treatment, the PA signal of the nanomotors is relatively weak; however, post-stimulation, the PA signal is markedly amplified. This amplification enables PAI to vividly display the location and composition changes of thrombi, thereby providing high-resolution images for thrombus diagnosis. Concurrently, nanomotors, with the support of color Doppler USI, can preliminarily determine the location of thrombi. When integrated with PAI, a comprehensive multimodal imaging system is formed, capable of thoroughly monitoring the formation and dissolution of thrombi. Furthermore, Fe_3_O_4_ NPs confer magnetic responsiveness upon nanomotors, enabling nanomotors to accurately accumulate at the thrombus site under the direction of an external magnetic field. This not only enhances the targeting of thrombolytic drugs but also improves the precision of imaging, thereby enabling multimodal imaging to more accurately reflect the conditions at the thrombus site.

In the realm of deep-tissue imaging, such as within the bladder, MNM-based multimodal imaging has witnessed substantial progress beyond dual-modal approaches. Li et al. [134] engineered a multifunctional *Spirulina*-derived hybrid helical microswimmer through a combination of intracellular biological deposition of AuNPs and surface assembly of magnetite nanoparticles (Fe₃O₄ NPs). The high atomic number of AuNPs confers exceptional x-ray absorption capability, yielding pronounced contrast in CT imaging. Leveraging this property, digital subtraction angiography (DSA) facilitates real-time dynamic tracking of the micromotors. For MRI, the superparamagnetic characteristics of surface-bound Fe₃O₄ NPs induce shortening of the T2 relaxation time of adjacent water molecules, thereby enhancing T2-weighted MRI signals. Intracellular AuNPs further modulate magnetic field homogeneity via charge transfer effects, contributing to improved contrast resolution. In the domain of PAI, the SPR of AuNPs at the NIR (808 nm) wavelength enables efficient conversion of absorbed light energy into thermoacoustic signals, with signal intensity demonstrating a concentration-dependent enhancement. Additionally, FI is achieved through retention of the native red autofluorescence of *Spirulina*. Although AuNP deposition induces a marginal reduction in fluorescence intensity, the remaining signal suffices for real-time superficial tissue tracking, providing complementary spatial resolution to other imaging modalities. This study presents a “surface modification–intracellular deposition” strategy to integrate magnetic micromotors with multimodal imaging capabilities (CT/DSA/MRI/PAI/FI), overcoming the functional monopathy of conventional micromotors and enabling precise deep-tissue navigation. Future investigations may focus on translating these findings into clinical applications for lumen-specific imaging, particularly in bladder tumor diagnostics.

## Intelligent System Implementation

In recent years, the rapid advancement of AI technology has led to remarkable breakthroughs. In particular, the convergence of motion control and AI in MNM-enhanced biological imaging has emerged as a cutting-edge research frontier within the domains of biomedical engineering and nanotechnology [135,136].

MNMs have established themselves as promising active imaging agents, offering unique advantages in navigating complex biological environments. By incorporating advanced trajectory optimization methods such as DL and reinforcement learning (RL), these miniature devices can be effectively guided through the intricate fluid dynamics and targeted requirements of living organisms [137]. RL algorithms enable MNMs to develop adaptive movement strategies: they can autonomously maneuver around obstacles, respond to biological stimuli (e.g., temperature and chemical gradients), and achieve precise localization of target regions. Through AI-driven dynamic optimization—including adaptive switching of propulsion modes and controlled release of contrast agents—these systems substantially enhance the specificity of lesion tissue imaging [138]. In complex dynamic environments, such as 3-dimensional vascular networks with obstacles and fluid disturbances, traditional motion control strategies for MNMs face critical limitations. While manual control remains feasible in simplistic scenarios, its inherent decision latency and poor environmental adaptability become pronounced in biologically relevant contexts. To address this gap, researchers are actively integrating machine learning and RL into navigation systems, aiming to boost both autonomous decision-making and real-time response capabilities.

To address the challenges of imaging in low-contrast and intricate backgrounds, Nauber et al. [139] introduced a dual-mode US and PAI system for real-time MNM imaging using DL technology. This model was meticulously fine-tuned using transfer learning and a custom dataset comprising various microrobot types, enhancing its detection prowess for diminutive targets amidst complex backdrops. The research further leveraged GPU acceleration to facilitate real-time beamforming and DL inference for ultrasonic/photoacoustic dual-modal imaging. By integrating precise synchronous imaging from the microcontroller with the time multiplexing technology of magnetic drive, they mitigated imaging artifacts due to electromagnetic interference, thus improving the overall imaging quality. Comparative analysis with optical tracking confirmed that this approach reduced the missed detection rate from over 91% in the ultrasonic threshold method to a mere 16.9% when combining photoacoustics and DL. The system achieved a root mean square error of 67.4 ± 15.3μm at a detection distance of 20 mm, offering an efficient and dependable imaging solution for real-time closed-loop control of microrobots in profound tissue environments.

**Fig. 14.**
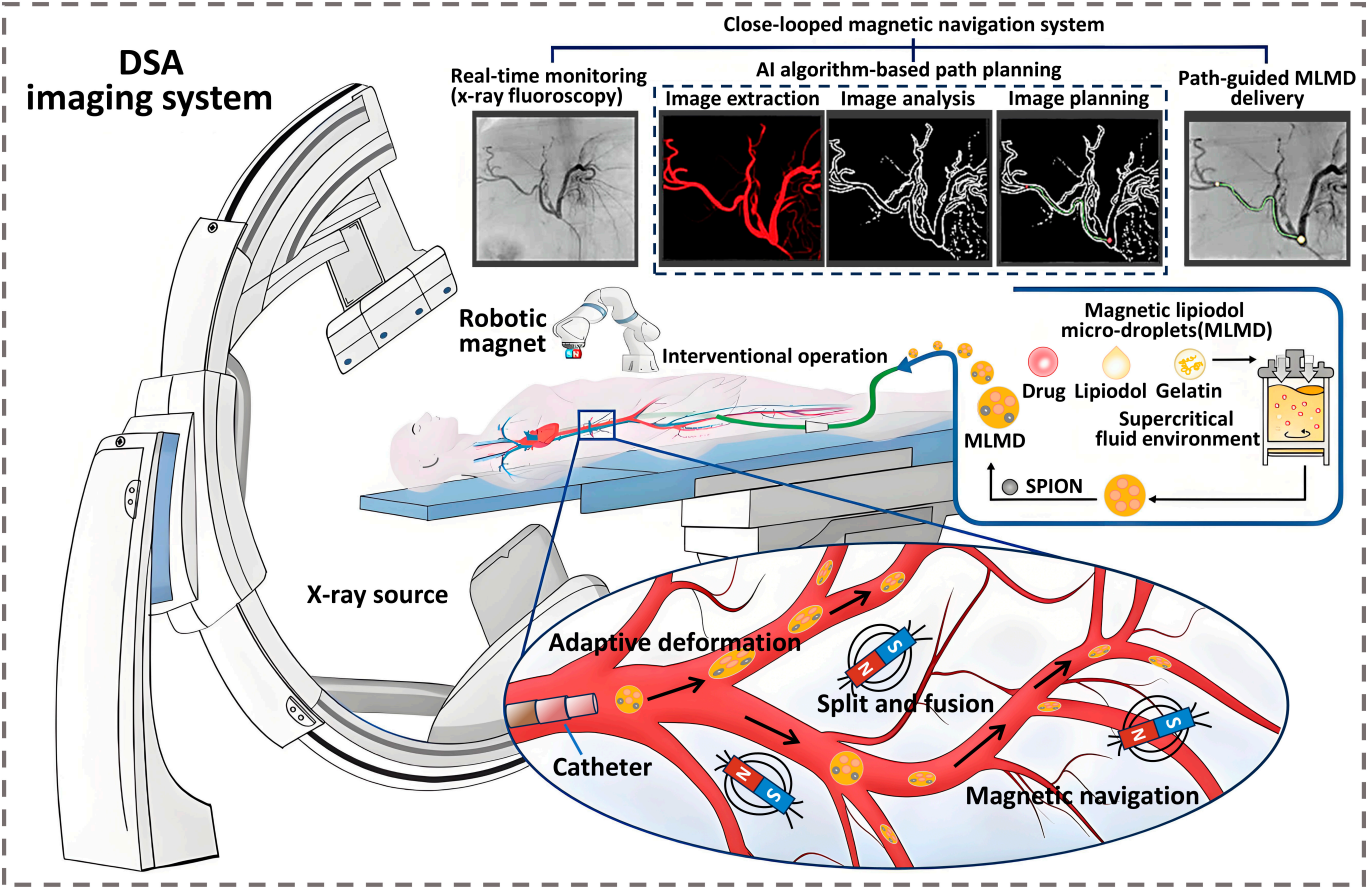
A closed-loop magnetic navigation system with artificial intelligence-driven visual feedback. This system can independently perform path identification, analysis, planning, and selection, effectively guiding MLMD to reach the destination along the predetermined drug delivery route [140], Copyright 2024 Wiley-VCH GmbH.

In order to achieve imaging enhancement within the body, the integration of AI with closed-loop magnetic navigation systems facilitates the imaging of MNMs. Ren et al. [140] employed computer vision algorithms, notably the Canny edge detection algorithm, to process captured images, subsequently generating binary matrices for accurate mapping of navigable paths and obstacle avoidance, as shown in Fig. 14. Target positions within the algorithm were ascertained using the You Only Look Once (YOLO)v5 object detection system. Path planning automation was realized by integrating the breadth-first search algorithm. Additionally, a proportional integral-derivative feedback control mechanism was implemented to modulate the magnetic field’s intensity and direction, ensuring precise control over the movement of water-stabilized magnetic iodized oil droplets. This mechanism tracked the positions of both the magnetic droplet and the magnet, computed subsequent speed adjustments for the magnet, and established a comprehensive closed-loop feedback system. It also employed affine transformation technology to translate the magnetic droplet’s position in the image to its absolute location on the magnetically controlled robot, thereby completing the feedback cycle. Through these techniques, AI enables the system to recognize, analyze paths, and navigate water-stable magnetic iodide oil microdroplets with precision. When combined with the inherent DSA imaging capabilities of these microdroplets, the system supports real-time monitoring and targeted drug delivery in intricate in vivo settings.

On the other hand, AI-assisted imaging analysis assumes a pivotal role in the MNM imaging system. By harnessing advanced deep-learning models, including convolutional neural networks (CNNs) and generative adversarial networks, it conducts super-resolution reconstruction and denoising on low-quality images. This not only enhances the overall image clarity but also enables precise focusing on critical regions within the images, such as the margins of tumors. As a result, the imaging quality is substantially improved, providing more detailed and accurate visual information for subsequent analysis and diagnosis. For instance, the Faster R-CNN algorithm utilizes the Region Proposal Network to generate multiscale anchor boxes, effectively tackling the challenges of scale variation and low contrast that MNMs present in ultrasonic images. In contrast, the Mask R-CNN algorithm excels at generating precise object masks, which enables it to extract intricate spatial features. By doing so, it resolves the issues of blurred edges and poor contrast associated with MNMs, markedly enhancing the detection precision for small targets [141]. Moreover, the integration of the YOLOv7 and Deep Simple Online Real-time Tracking algorithms offers a viable solution for extracting depth and pose information of MNMs from imaging data [142]. By leveraging these advanced algorithms, the inherent limitations of planar images in conveying comprehensive information can be effectively mitigated, thereby enabling more accurate and detailed characterization of imaging targets.

## Summary and Outlook

In the emerging domain at the intersection of nanotechnology and biomedicine, MNMs are proving to be pivotal. These miniature devices, which convert external energies such as chemical, electrical, and light into directional motion, offer a unique set of applications. This presents an innovative approach to overcoming the limitations of conventional biological imaging technology. These micro-devices can autonomously navigate within organisms, pinpoint specific tissues or cells, and execute dynamic monitoring in complex physiological environments. Consequently, they usher in a new era in the realm of biological imaging. This paper explores the utilization of MNMs in biological imaging, detailing the benefits of this innovative biomimetic material and its potential future applications in precise imaging. Despite substantial advancements, several formidable challenges persist in the field of MNMs-enabled imaging.

### Motion-related challenges

The motion behavior of MNMs is a core parameter for enhancing the efficacy of bioimaging techniques. High-speed motion enables the motors to quickly overcome biological barriers, navigate efficiently to the target area, substantially shorten the enrichment time, and increase the local contrast agent concentration, thereby enhancing the imaging signal intensity and contrast. At the same time, the fluid shear force generated by the motion can reduce nonspecific adsorption, effectively suppress background noise, and improve the signal-to-noise ratio. In addition, speed endows the motors with the ability to actively penetrate deep tissues and supports them to act as mobile imaging probes for dynamic scanning, indirectly promoting the improvement of temporal–spatial resolution. The locomotive efficiency of MNMs operating within biological media remains a critical bottleneck. The high viscous resistance and intricate physiological milieu of these media substantially hamper the propulsion velocity of MNMs, thereby rendering deep-tissue imaging an arduous task. Leveraging AI-driven design frameworks to engineer MNMs with optimized kinematic structures holds promise for surmounting these limitations, facilitating high-resolution imaging of deep-seated tissues.

### Control-related challenges

The motion control of MNMs is a crucial guarantee for achieving precise, efficient, and safe imaging. The synergy between real-time feedback control and external fields can optimize the spatiotemporal behavior of motors during the imaging process (such as synchronizing the imaging frame rate and motion trajectory), enabling the monitoring of dynamic physiological processes and minimizing motion artifacts. Therefore, motion control not only determines whether MNMs can translate their autonomous motion advantages into usable imaging enhancement efficiency but also serves as the core hub for balancing targeting accuracy, penetration depth, signal-to-noise ratio, and biosafety. There exists an acute demand for highly precise motion control methodologies for MNMs, accompanied by the imperative to augment their targeting accuracy. Future developments in AI-integrated closed-loop control systems are anticipated to introduce intelligent features, such as autonomous identification of imaging loci, followed by the strategic planning of imaging agent trajectories. This would enable the realization of highly targeted and precise imaging modalities.

### Application-related challenges

From an application standpoint, current single-modality MNMs-enhanced imaging techniques fall short of fulfilling clinical requisites. In vivo clinical applications necessitate imaging technologies capable of capturing multidimensional information. Going forward, the fabrication of multimodular MNMs represent a key research direction. Such motors could facilitate the diversification of MNM-based imaging functions and the integration of multidimensional information-rich imaging paradigms. Additionally, ensuring the biocompatibility and degradability of MNMs materials poses a substantial hurdle. Mitigating the biotoxicity of these motors to negligible levels and developing integrated systems that seamlessly combine imaging, diagnostic, and therapeutic functionalities are essential for translating MNM technologies into clinical practice.

Despite the formidable challenges outlined above, researchers are poised to not only formulate and meticulously refine the theoretical frameworks but also enhance and optimize experimental methodologies. Through these concerted efforts, a robust foundation will be meticulously constructed, while innovative technologies will be developed. These advancements will serve as a cornerstone for the burgeoning applications in diverse fields, particularly in the realm of precise biological imaging facilitated by MNMs, thereby propelling the frontiers of scientific research and technological innovation in this domain.

## Data Availability

No new data were created or analyzed during this study. Data sharing is not applicable to this article.
